# Study protocol for a hybrid implementation-effectiveness trial of Game Changers for Cervical Cancer Prevention in Uganda

**DOI:** 10.1371/journal.pone.0317491

**Published:** 2025-01-24

**Authors:** Glenn J. Wagner, Laura M. Bogart, Joseph K. B. Matovu, Violet Gwokyalya, Jolly Beyeza-Kashesya, Allison Ober, Harold D. Green, Sylvia Nakami, Margrethe Juncker, Eve Namisango, Emmanuel Luyirika, Ryan K. McBain, Kathryn Bouskill, Rhoda K. Wanyenze

**Affiliations:** 1 RAND Corporation, Santa Monica, California, United States of America; 2 Department of Medicine, Charles R. Drew University of Medicine and Science, Los Angeles, California, United States of America; 3 School of Public Health, Makerere University, Kampala, Uganda; 4 Busitema University Faculty of Health Sciences, Mbale, Uganda; 5 Mulago Specialized Women and Neonatal Hospital, Kampala, Uganda; 6 School of Medicine, Makerere University, Kampala, Uganda; 7 Indiana University School of Public Health, Bloomington, Indiana, United States of America; 8 Rays of Hope Hospice Jinja, Jinja, Uganda; 9 African Palliative Care Association, Kampala, Uganda; PLOS: Public Library of Science, UNITED KINGDOM OF GREAT BRITAIN AND NORTHERN IRELAND

## Abstract

**Introduction:**

Cervical cancer (CC) is the leading cause of cancer-related deaths among Uganda women, yet rates of CC screening are very low. Training women who have recently screened to engage in advocacy for screening among women in their social network is a network-based strategy for promoting information dissemination and CC screening uptake.

**Methods:**

Drawing on the Exploration, Preparation, Implementation and Sustainment (EPIS) framework for implementation science, this hybrid type 1 randomized controlled trial (RCT) of a peer-led, group advocacy training intervention, *Game Changers for Cervical Cancer Prevention (GC-CCP)*, will examine efficacy for increasing CC screening uptake as well as how it can be implemented and sustained in diverse clinic settings. In the Preparation phase we will prepare the four study clinics for implementation of GC-CCP and the expected increase in demand for CC screening, by using qualitative methods (stakeholder interviews and client focus groups) to identify and address structural barriers to easy access to CC screening. In the Implementation phase, GC-CCP will be implemented over 36 months at each clinic, with screened women (index participants) enrolled as research participants receiving the intervention in the first 6 months as part of a parallel group RCT overseen by the research study team to evaluate efficacy for CC screening uptake among their enrolled social network members. All research participants will be assessed at baseline and months 6 and 12. Intervention implementation and supervision will then be transitioned to clinic staff and offered as part of usual care in the subsequent 30 months as part of the Sustainability phase. Using the RE-AIM framework, we will evaluate engagement in GC-CCP and CC advocacy (reach), alter CC screening (effectiveness), adoption into clinic operations, implementation outcomes (acceptability, feasibility, fidelity, cost-effectiveness) and maintenance.

**Discussion:**

This is one of the first studies to use a network-driven approach and empowerment of CC screened peers as change agents to increase CC screening. If shown to be an effective and sustainable implementation strategy for promoting CC screening, this peer advocacy model could be applied to other preventative health behaviors and disease contexts.

**Trial registration:**

NIH Clinical Trial Registry NCT06010160 (clinicaltrials.gov; date: 8/17/2023).

## Introduction

Cervical cancer (CC) is the leading cause of cancer-related deaths among Uganda women, yet rates of CC screening are very low. CC accounts for ~25% of all cancer deaths in Ugandan women, [1–3] and over 80% of women presenting for care have advanced disease [4]. Treatment for CC is available at only one facility in the country and is too costly for vast majority of women. This highlights the importance of timely, periodic screening, and early-stage treatment (for pre-cancerous lesions) when warranted, both of which are evidence-based standard of care, [5, 6] as well as free or low cost in many part of Uganda. Yet the lifetime screening rate for CC in Uganda is 5–15%, despite a human papillomavirus (HPV) prevalence of 34%, [4, 7, 8] recommendations for CC screening every 3 years, and the World Health Organization (WHO) global strategy to accelerate elimination of CC by striving for a 70% screening rate [9].

Structural barriers (i.e., inner and systems setting) to CC screening include limited access (screening is not available in all districts), cost of transport, and lack of client education from providers [10, 11]. Non-structural barriers (i.e., outer setting) include low awareness and misinformation, fear of results, and stigma due to CC stemming from a sexually transmitted infection and symptoms of advanced disease that lead women to be shunned and isolated [11–14]. Facilitators of screening that have been identified are outreach education, support and encouragement from others, and knowing someone who has been screened or diagnosed [7, 11, 13].

Network-based, peer advocacy interventions in the literature [15, 16] draw on network diffusion theory [17] and principles of social identity and social influence [18] to posit that behavior change can be initiated by a few and diffused to others through modeling, advocacy, and shifts in norms [19, 20]. Intervention effects on increased advocacy, [21] and improved health behavior of the recipients of advocacy, [22, 23] have been observed in the context of HIV risk and drug use. In the context of CC, peer education has been evaluated in healthcare workers, [24] male partners, [25] and women at risk, [13, 14] but not through clients’ social networks. Drawing on these theories and evidence-based peers advocacy interventions, and knowledge of the non-structural barriers and facilitators of CC screening described above, we developed the peer advocacy intervention *Game Changers for Cervical Cancer Prevention* (GC-CCP) to increase CC screening uptake.

**Fig 1:**
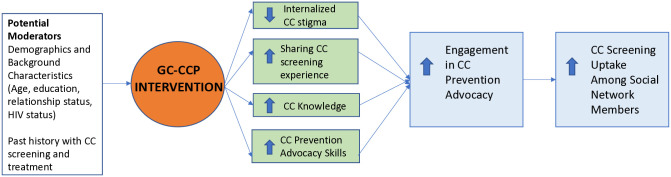
Conceptual framework for the Game Changers Cervical Cancer Prevention (GC-CCP) peer advocacy training intervention and its promotion of cervical cancer (CC) screening among social network members.

GC-CCP empowers women who have recently screened for CC to encourage screening among women in their social networks. The conceptual framework of the intervention and its content target stigma reduction, sharing of CC screening experience, CC knowledge, and advocacy skills for supporting behavior change (see Fig 1). *Effective advocacy first requires coping with fears and internal stigma* [26, 27]. Coming to terms with one’s CC diagnosis or risk fosters self-acceptance and self-compassion, and is related to received stigma, discrimination and support [28, 29]. Self-acceptance facilitates *sharing one’s CC screening experience*, which enhances the credibility of one’s advocacy for others to get screened. However, disclosure of CC risk can increase support as well as lead to rejection or ridicule, and thus disclosure decision-making skills are important. For effective advocacy, women must *learn the basic facts and myths related to cervical cancer and screening*, so that they have the knowledge to engage in accurate advocacy and to address misconceptions that peers express. *Learning communication skills for who*, *when and how to engage in advocacy* are key to effective advocacy.

In a pilot randomized controlled trial (RCT) of GC-CCP, 40 women who had screened for CC (index participants) enrolled and were assigned to receive the peer-led, multi-session group intervention or the wait-list control, and 103 of their social network members (referred to as “alters”) who had never previously screened for CC were also enrolled. All were followed for 6 months, and a strong intervention effect was found on alter CC screening (64% among alters in the intervention arm vs. 16% of those in the control group) [30]. Furthermore, CC prevention advocacy increased significantly among both the index and alter participants in the intervention arm compared to their control counterparts, suggesting a diffusion of advocacy effect within the network [31, 32].

To further establish the efficacy of the intervention and assess how it can be implemented and sustained in diverse settings, we are conducting a hybrid randomized controlled trial of GC-CCP. Guided by the Exploration, Preparation, Implementation and Sustainment (EPIS) implementation science framework, [33, 34] the study will first identify and remediate structural barriers to easy access to CC screening at the study sites. This will be followed by a 3-year implementation of the intervention, initially by the study team to assess efficacy in increasing CC screening (6 months), and then by the clinic staff to assess sustainability (30 months). Using the RE-AIM (Reach, Effectiveness, Adoption, Implementation, Maintenance) framework for implementation science, [35] we will evaluate engagement in the group intervention and CC advocacy (reach), alter CC screening (effectiveness), adoption into clinic operations, implementation outcomes (acceptability, feasibility, fidelity, cost and cost-effectiveness), and maintenance over time. The primary objectives of the study are to (1) conduct a multisite RCT of the GC-CCP network-based advocacy strategy to evaluate effects on CC screening uptake among unscreened alters; (2) use a mixed methods approach (semi-structured interviews and administrative clinic data) to examine clinic-, provider-, and client-level barriers and facilitators of GC-CCP implementation and sustainment; and (3) evaluate the cost-effectiveness of GC-CCP to increase CC screening compared to usual care.

## Methods

### Study design

Drawing on the EPIS framework, [33, 34] this hybrid type 1 RCT will be conducted in three phases to evaluate GC-CCP for increasing CC screening, and identify barriers and facilitators to sustained implementation across diverse clinic settings. We have used the SPIRIT reporting guidelines to document the study methods in the study protocol [36]. The Exploration phase to assess the feasibility and acceptability of GC-CCP, including potential barriers and facilitators to the intervention as well as CC screening, was completed during the pilot trial of GC-CCP [30]. The current study continues at the Preparation phase: to prepare the four study clinics [two public and two private-not-for-profit (PNFP), and one of each in an urban and rural location] for implementation of GC-CCP, we will identify modifiable clinic and provider level barriers to easy access to CC screening and conduct feasible remediation of selected barriers. In the Implementation phase: at each clinic, GC-CCP will be implemented over 36 months, with women enrolled as research participants receiving the intervention in the first 6 months as part of a parallel RCT overseen by the research study team to evaluate efficacy. Implementation and oversight of the intervention will then be transitioned to clinic staff and female clients will receive the intervention on a quarterly basis as part of usual care in the subsequent 30 months as part of the Sustainability phase. Using the RE-AIM framework, [35] we will evaluate engagement in GC-CCP and CC advocacy (reach), alter CC screening (effectiveness), adoption into clinic operations, implementation outcomes (acceptability, feasibility, fidelity, cost and cost-effectiveness) and maintenance.

### Study setting

The trial will be conducted in four clinics—Nsambya Hospital (PNFP) and Kawempe National Referral Hospital (public) in urban Kampala, and St. Charles Lwanga Hospital (PNFP) and Kayunga Regional Referral Hospital (public) near the rural town of Jinja. The sites vary by urbanicity and funding source, external factors that may influence challenges related to GC-CCP implementation and sustainment (e.g., client awareness and attitudes towards CC screening; availability of clinic resources). Also, public and PNFP clinics are each utilized by about 40% of the Ugandan population, [37] so representation from both sectors bolsters the study’s external validity. All sites offer CC screening using visual inspection with acetic acid (VIA), and most offer human papillomavirus HPV screening for HIV-infected clients only. All but one site offers thermocoagulation for treatment of pre-cancerous lesions. These CC-related services are provided by 2–4 trained staff, depending on the site. All but the St. Charles Lwanga Hospital conduct biopsies and pap smears (for menopausal women), but none provide treatment for CC; women are provided referrals to Uganda Cancer Institute in Kampala for such treatment.

### Phase 1: Preparation

Since the GC-CCP intervention is expected to increase demand for CC screening, it is important that screening be as accessible as possible at the study sites. To prepare the sites for implementing GC-CCP, Year 1 of the study will focus on identification and remediation of clinic and provider level barriers to easy access to and delivery of CC screening. Across the four study sites, we will conduct 30 ***semi-structured in-depth interviews*** with providers, health officials and policymakers, and 8 ***focus groups*** (2 per clinic) with CC screened and unscreened clients. All participants will be given 40,000 Ush (~$10). For the in-depth interviews, an interview guide based on EPIS domains [33, 34] and recommendations for implementation research outcomes (appropriateness, acceptability, feasibility) [38] will be used (see Table 1). Interviews will start with a general “grand-tour” questions, followed by more focused questions to elicit in-depth information about provider and clinic barriers to CC screening and GC-CCP implementation, proposed solutions to barriers, and feedback on the program’s fit with the clinic [39]. The focus group guide will focus on the acceptability of and barriers to CC screening and engagement in GC-CCP. Interviews and focus groups will be audiotaped, transcribed, and translated. Using standard analysis methods, [40, 41] two team members will read all transcripts to develop a list of themes for the implementation constructs, and a codebook listing each theme accompanied by a detailed description, inclusion/exclusion criteria, and examples. Using Dedoose software, two coders will mark text corresponding to each theme. Coders will independently code a randomly selected 20% of transcripts to assess coder consistency (i.e., Kappa ≥.70). We will examine distribution of themes, overall and by participant type [42].

**Table 1:** Sample provider semi-structured interview guide.

EPIS Domain	Construct	Sample Questions/Measures
**Bridging Factors**	* Leadership support	* To what extent does your clinic leadership support CC screening? *How does that affect implementation*?
		* To what extent are [have] you been you willing to work on a project with researchers from outside of the clinic? *How might [does] research affect clinic workflow*?
	* Relationships	
		* What partnerships (e.*g*., *with NGOs) typically help or hinder implementation of practices at this clinical generally*? *CC screening specifically*?
		* What other relationships in/out of this clinic [could] help or hinder CC screening [GC-CCP] implementation?
**Innovation**	* Effectiveness	* How effective do you think CC-screening is [or will be]? *GC-CCP*?
	* Complexity	* How easy or hard is the process of conducting CC screening? *GC-CCP*?
	* Relevance/Fit	* How does CC screening fit with your work duties? *GC-CCP*? *How might they detract*?
	* Acceptability	* What are your perceptions of the feasibility of CC screening? *GC-CCP*?
**Inner Context**	* Provider Knowledge	* How confident are you in conducting CC screening? *GC-CCP*? *Why/why not*?
		* How willing are you to conduct CC screening [GC-CCP] with all female patients? *Why/why not*?
	* Provider Self-efficacy	* How prepared (or not) are you to conduct CC screening [GC-CCP] for all female patients? *Why/why not*?
	* Provider Motivation	* How much have you had to turn women away who wanted to be screened? *Why*?
		* What challenges might/did affect implementation of GC-CCP in your clinic?
		* How do you think that GC-CCP could/did affect clinic flow? *…increase your workload*?
	* Appropriateness	* How much is staffing/resources an issue? *How could these barriers be addressed*?
	* Feasibility	* What kind of [added] training do you think GC-CCP peer facilitators need?
**Outer Context**	* Local /national regulations and requirements	* How do Ministry of Health policies affect implementation of CC screening here?
		* How do funder policies and requirements affect CC screening?
		* What other local or national policies affect CC screening? *How so*?
	* Client characteristics	* How open are your patients to CC screening? *GC-CCP participation*?
		* What patient factors impede CC screening? *Participation in GC-CCP*?
**Other**	* Other barriers	* What other factors affect CC screening here? *GC-CCP implementation*?
		* What main things that need to change for all clients to be CC screened? *GC-CCP*?
		* What can be done to make sure CC screening [GC-CCP] continues at this clinic?

With the interview and focus group data, we will compile a list of barriers and gaps, then with leadership at each clinic we will use ***Plan-Do-Check-Act (PDCA) cycles*** [43–46] to address primary selected barriers. PDCA cycles offer a structured plan to engaging staff in making iterative, feedback-based changes in service delivery, which help to ensure that changes fit within an organization and can lower resistance from providers who will be affected by changes in delivery [47]. Planning: We will work with the health facility leadership and all staff involved with CC service delivery at each site to form Core Teams of 6–8 persons drawn from across relevant departments, and together with the Core Team we will conduct a situational assessment. This assessment includes brainstorming on the “process-related” challenges affecting CC screening access and uptake at their site, coming up with a general list of problems (using data from the in-depth interviews and focus groups as a starting point from which to add to) and selecting main problems to address, identifying the root cause of that problem, and generating strategies to address the problems within 6–9 months. Doing: Site teams will implement the countermeasures over a period of 6–9 months. Checking: The Core Team will evaluate the changes made in access to and uptake of CC screening services at the end of this period. Acting: Additional modifications will be made to address ongoing barriers and gaps. At the end of the Preparation phase, we will have identified mechanisms through which improvements can be achieved, and we will have created quality improvement teams at each site that we will work with during the Implementation phase.

To ensure a comparable environment of access to CC services across all sites by the completion of the Preparation phase, the project will provide thermocoagulators (and any necessary provider training on thermal therapy) for the site that currently does not offer this treatment, as well as other equipment or supplies needed for optimal client access to CC screening and thermal therapy. Once the Implementation phase begins, the sites will be responsible for ensuring an adequate, sustainable stock of supplies, so that the study can assess the ability of the sites (and external stakeholders, such as Uganda Ministry of Health) to manage this critical factor over time. In addition, providers in all departments at each site will participate in a 2-hour continuing medical education (CME) onsite training that will provide information on CC etiology, epidemiology, prevention and treatment.

### Phase 2: Implementation

Following the one-year Preparation Phase, the GC-CCP intervention will be implemented over three years. In the first six months of the GC-CCP implementation, the hybrid type 1 effectiveness-implementation RCT begins, and enrolled index participants receive GC-CCP. A hybrid type 1 trial [48] tests “a clinical intervention while gathering data on its delivery during the effectiveness trial, and its potential for implementation in a real-world situation.” The hybrid trial will collect effectiveness and implementation data, but the RCT will focus mostly on evaluating the intervention’s effectiveness in increasing alter CC screening. However, other implementation outcomes such as reach (client enrollment in the GC-CCP training, and index and alter engagement in CC screening advocacy) will draw on data from the research cohort enrolled in the RCT, but these outcomes will be more fully evaluated in the Sustainability phase. Recruitment for the RCT began August 20, 2024 and is expected to be completed in August 2025. Data collection for the RCT is expected to be completed in February 2026. Results from the RCT should be fully reported by January 2027.

#### Game Changers for CC Prevention (GC-CCP)

GC-CCP consists of six sessions to empower CC screened women to act as change agents for CC screening in their social networks. ***Session 1*** addresses fears and concerns related to CC risk and use of self-compassion and peer support to overcome these fears. ***Session 2*** focuses on decision making for sharing one’s personal CC screening experience, and how to initiate disclosure and conversations about CC. ***Session 3*** focuses on CC facts and myths, and helping participants address misconceptions about CC during discussions with others. Participants also think about the women in their social network and identify women who may be strategic targets for CC advocacy. ***Sessions 4 to 6*** build CC advocacy skills such as communication skills (e.g., reflective listening, paraphrasing, open ended questions) and how to start and sustain conversations about CC. These sessions aim to inspire commitment to ongoing CC advocacy through peer solidarity and support. The 2-hour, weekly sessions will be conducted in Luganda using a structured manual, and group format to facilitate: sharing of experiences to build solidarity, support, and motivation among participants; group problem solving and role playing to build skills and self-efficacy; and homework between sessions to practice new skills and generate experiences to be processed in the sessions.

*Facilitator training*, *supervision*, *and fidelity monitoring*. Two bilingual (Luganda, English), literate female clients of the clinic will be recruited by the clinic staff and trained as intervention peer facilitators. Criteria for facilitators include having in-depth knowledge of the local community and personal experience of being screened for CC; they may be counselors or community health workers (trained lay persons) whose role often includes community outreach and education, and facilitating client engagement and use of health services. The training, conducted by the study’s senior investigators, will include reviewing objectives for each session, step-by-step scripts, role playing and mock implementation of core exercises. Training will cover group facilitation, building rapport, reflective listening, and dealing with group conflict. The supervisors of the initial two groups at each site (i.e., the groups administered to the research index participants) will attend each session and complete fidelity rating forms; each facilitator will also complete fidelity forms after each session. These forms will include ratings of fidelity to the manual, participant engagement, and quality of facilitation, and will be used to facilitate weekly supervision between sessions.

#### RCT evaluation of CC-CCP

**Fig 2:**
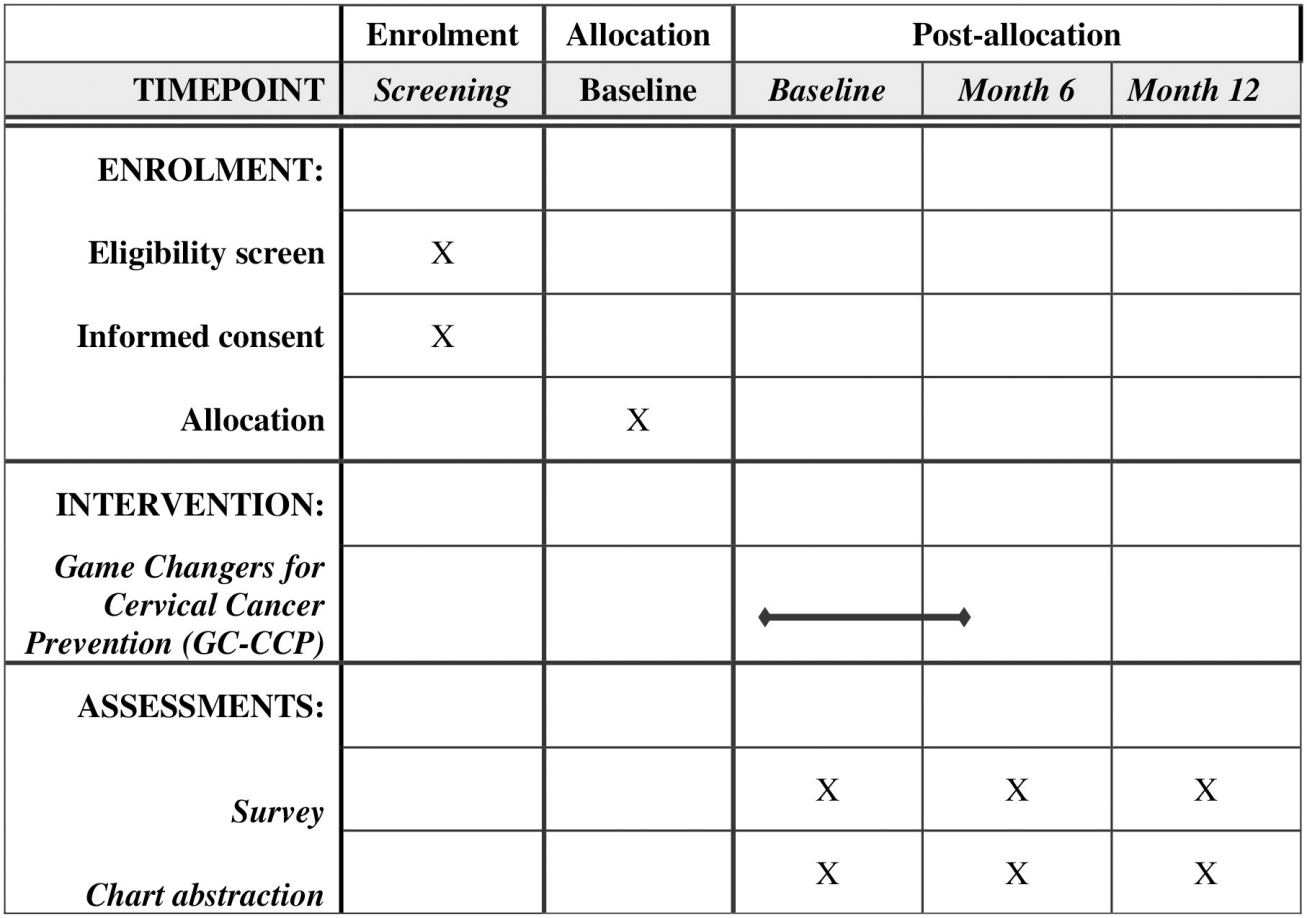
SPIRIT guideline checklist.

At each of the four sites, 40 women screened for CC in the past year will enroll as index participants (n = 160), with 20 randomly assigned to the intervention, and 20 to a wait-list usual care control. The intervention arm will be divided into two groups of 10 (balanced on ages 18–35 and 36+) for receipt of the intervention. The control arm receives the intervention after the 12-month follow-up assessments have been completed. There are no specific criteria for discontinuing or modifying allocated intervention condition for individual participants. A non-intervention control was used, as opposed to an attention control that would focus on the intervention participants, as this attention would not be comparable to the control for attention needed for effects on social network members (who provide data for the primary hypothesized outcomes). Participants in both groups receive care as usual from the study site, which does not involve any training related to advocacy. Each index participant will recruit up to three alters (maximum total = ~440 alters; referred to as first degree alters) with no history of CC screening. All index and first degree alter participants will be followed up at months 6 and 12. At month 6, first degree alters will recruit up to two of their own alters (second degree alters; total = ~800); second degree alters will be administered a single, brief phone interview. Fig 2 outlines the schedule of trial activities, including assessments and intervention sessions.

*Eligibility criteria*. *Index participants*: female, age 18 and older, screened for CC in past year (verified by clinic records), and no advanced stage disease (12-month follow-up is feasible). *First degree alters*: female, age 18 and older, and report no CC screening history. *Second degree alters*: female and age 18 and older.

*Recruitment*. Clinic staff will contact eligible women who have been screened for CC in the past year to inform them of the study; if interested, the site coordinator will perform consent processes and formal eligibility screening. Eligible women who enroll will complete the baseline survey, followed by randomization as an index participant. First degree alters: The site coordinator will use the social network assessment within the baseline survey of each index to randomly select (using a random number table) five alters who know the participant’s CC screening experience (or as many as there are if < 5) and ask the index if she is comfortable recruiting three of these alters. The index will be asked to call each selected alter at the end of their baseline interview to describe the study in the presence of the coordinator (or later in private, if she prefers), who will then schedule a study visit for the alter. If an alter refuses or cannot be reached, a replacement will be randomly chosen from the original list of five alters. Second degree alters: The site coordinator will use the social network assessment in the month 6 survey of each first degree alter to randomly select four alters (or as many as there are if < 4) who the alter reports targeting with CC screening advocacy and thereafter was screened for CC. A similar process as described above for first degree alters will be used to recruit and enroll second degree alters. The coordinator will obtain verbal consent from the second degree alter over the phone and then conduct a brief phone survey.

The consent process will include information about how a version of the study data in which all identifying information is removed may be made available for use by other scientists for the purpose of further research. Given the nature of the intervention, there is no anticipated harm. Informed consent materials are available, on request, from the corresponding author. To promote study retention, we will collect tracking information (phone numbers, mapped addresses, contacts for family/friends with whom they have frequent contact).

*Randomization*. A blocked 1:1 randomization design with stratification by age (age 18-35/36+ years) and screening result (positive/negative) will be used with randomly alternating blocks of 2, 4, and 6 to prevent anticipation of assignment to ensure balance across arms. The study statistician will use a random number generator to devise a sequential randomization code list that will be used by the study coordinator to assign index participants to the intervention or control arms. The coordinator and data collectors are blind to the assignment until after the baseline assessments are completed; after the random assignment is revealed, the only blind party is the data analyst.

*Assessment schedule*. Surveys will be administered at baseline, month 6 and month 12 for index and first degree alters, while second degree alters will be surveyed only once (at month 6 assessment of first degree alters). Participants will receive 30,000 Ush (~$8) per assessment, except second degree alters who will receive 10,000 Ush (via mobile money). To limit attrition, we will collect tracking information, including contacts for family/friends with whom they have frequent contact. Clinic staff will help us track clients we have difficulty reaching.

*Measures*. Survey assessments for index and first degree alter participants will be in-person and interviewer-administered in Luganda using Network Canvas software. These interviews will last approximately 75 and 30 minutes, respectively and include a social network assessment. Second degree alters will receive one 15-minute phone interview. CC screening and treatment utilization will be verified with medical chart data. All measures will be assessed at each of three time points.

*Primary outcome*: *CC screening*: Participants will self-report utilization of all CC-related services including screening (VIA, HPV, pap smear), treatment (thermal or cryo-therapy; radiation, chemotherapy, surgery) and biopsies. Any reported utilization will be verified with abstraction of medical chart data.

*Secondary outcome*: *engagement in CC prevention advocacy*: This will be assessed with participant self-report, both in terms of frequency of general advocacy with respect to specific CC-prevention topics, and more specifically with CC screening advocacy targeted at alters named in their social network assessment (described below).

Other constructs that will be assessed include targets of the intervention that are potential mediators of the intervention effect. These include internalized CC stigma, sharing of CC experience, CC knowledge, and advocacy self-efficacy, all of which will be assessed with participant self-report using measures we developed and validated in our prior research [30–32].

*Social network assessment*. The participant will list 10 female alters with whom they interact most. For each listed alter, we will gather information to assess *network composition* (e.g., age, relation to respondent, history of CC screening and treatment; knowledge of respondent’s CC screening experience). In the index survey only, we will ask how if each alter knows each other alter to assess *network structure* (e.g., density); this section is time consuming, so given the large number of alter participants, alters will not complete this survey component. *To assess CC advocacy and perceived effects*, we will ask the participants if they have engaged in CC screening advocacy (separate items for discussed, encouraged, gave info, gave direct support, and frequency) with the specific alter in the past 6 months, and any perceived resulting action (e.g., alter was screened). At follow-up, we will determine whether listed alters are the same or unique from those listed at baseline, to facilitate longitudinal analyses of alter data. Enrolled alters of the index participant will be asked about advocacy they received from the index to gain their perspective on the characteristics and impact of the advocacy.

*Data Analysis*. *Power*: The power analysis was calculated based on the primary outcome. Assuming 220 first degree alters per arm enroll, 10% attrition, intraclass correlation (ICC) between .01 to .05 to control for clustering within the alters of each index participant, and a 15% CC screening rate in control alters (based on our pilot data): the effective sample size will be 200 (ICC = .05) or 216 (ICC = .01) per arm. If ICC = .01, we will be able to detect a small effect size (11 percentage point difference) in alter CC screening at month 12 (80% power; alpha = .05). If ICC = .05, the detectable effect size is 12 percentage point difference (or odds ratio of 2.0).

*Assess effects of GC-CCP on alter CC screening*: We will use an intent-to-treat approach. In addition to comparing the arms at baseline and months 6 and 12, we will apply logistic generalized mixed models to our repeated-measures data to examine intervention effects, using a time by arm interaction to assess differences between the arms over time. We will account for correlation among participants in the same intervention group, and among alters referred by the same index, by adjusting standard errors for statistical inference tests with a sandwich estimator. We will use imputation for item nonresponse and attrition weights to account for non-random dropouts using logistic regression. We will control for and examine interaction effects with index (e.g., age), alter (e.g., knowledge of index’s CC screening), and intervention (sessions completed) variables.

*Evaluate the cost and cost-effectiveness of implementing GC-CCP to increase CC screening*: We will compare GC-CCP vs. usual care on the marginal cost of increasing alter CC screening. Following standard convention, [49] we will define the incremental cost-effectiveness ratio (ICER) as the difference in per-capita cost of the intervention versus control group divided by the difference in their average effectiveness: $ICER=\frac{\mu_{C2}-\mu_{C1}\;}{\delta_{e2}-\delta_{e1}}$ (μ_c2_ = mean per-capita cost of GC-CCP, μ_c1_ = mean per-capita cost of usual care, δ_e2_ = % of alters who get screened in GC-CCP, δ_e1_ = % of alters who get screened in usual care. We will estimate confidence intervals with bootstrap methods [50]. We will examine economic costs from a societal perspective, incorporating estimates of the frequency and duration of time spent by individuals engaging in receipt of services, as well as transportation costs and forgone employment as relevant. We will use a micro-costing approach, time-driven activity based costing, to track all costs associated with implementing GC-CCP and usual care as estimated from data collected by the sites and study team [51]. The cost per resource will be calculated by multiplying the quantity used by unit cost; total cost will be derived by summating individual costs [52]. Capital costs will be annualized using a discount rate of 3% with an assumed lifespan of 30 (buildings) and 10 (furniture) years [53]. Facilitators will record time spent on the sessions, identifying each activity (e.g., training, preparation, in-session) and session-related materials (e.g., consumable materials). Unit costs for labor will be characterized as capacity cost rates. Intervention training and ongoing costs will be differentiated; ongoing costs will be tracked to determine cost efficiencies over time, and we will differentiate fixed from variable intervention costs to assess the marginal cost of providing care to additional clients.

### Phase 3: Sustainment

#### Evaluation of ongoing GC-CCP implementation and maintenance

After implementing GC-CCP with research enrolled index participants in the first 6 months of implementation to evaluate effectiveness, clinic staff will take over all aspects of implementation during the Sustainment phase (2.5 years) of implementation. The study team will transition the training and supervision of peer facilitators to clinic providers. This phase is important to (a) fully understand implementation processes, and facilitators of and barriers to real-world implementation of GC-CCP (without research staff); (b) to study sustainability of GC-CCP (as well as CC prevention advocacy and CC screening) when GC-CCP is fully implemented by clinic staff; and (c) to prepare the clinics to maintain GC-CPP into the future, if it is found to be cost-effective.

*Identifying and training clinic staff to supervise GC-CPP*. The study team will work with the clinics to identify suitable persons to serve as supervisors for the Sustainment phase. The selected staff member will likely vary from site to site based on variability in staff composition and skill sets, but female counselors or volunteer lay persons who assist in the clinic may be suitable, if they have experience supervising others. Nurses are not preferrable in this role given that they are already overburdened in these busy clinics. The peer facilitators will be identified and trained at the start of the Implementation phase for the RCT, but there is likely to be some turnover and need for training of new facilitators during the 3-year implementation period. The person who will train and supervise the peer facilitators will be identified at the start of the RCT; this will enable them to participate in the initial facilitator training, and to shadow the supervisors from the study team as they carry out supervision during the RCT. In the initial supervision sessions conducted by the staff member in the Sustainment phase, one of the study team supervisors will attend these sessions to provide continued mentorship. Supervision will be held after each session for the first group they oversee, followed by after every other session.

RE-AIM outcomes will be assessed to evaluate GC-CCP implementation, as operationalized in Table 2. ***Effectiveness*** for increasing CC screening (primary RCT outcome) will be based on self-report and medical chart abstracted data from alter participants enrolled in the RCT. ***Reach*** in terms of how many women engage in GC-CCP and CC prevention advocacy will be assessed using chart data from CC screened clients, and survey data from enrolled index and alter participants, respectively. At the beginning of the RCT (Implementation Phase), we will work with the clinic to add a field to the client visit form to record referral source for CC screening [e.g., healthcare provider, peer (family, friend or other client)]; and if relevant, RCT enrollment (and date) and participation in GC-CCP (dates of sessions attended). Follow-up survey data from enrolled index and alter participants in the RCT will inform ***Maintenance*** in terms of sustained engagement in CC prevention advocacy. Semi-structured interviews and surveys with clinic staff and clients, supervisor ratings of facilitators (fidelity), and clinic budget ledgers (cost), will be used to assess EPIS constructs [33, 34].

**Table 2:** RE-AIM outcomes.

Outcome	Operationalization	Data Source & Study Phase
**Reach** (CC screened women)	a) Proportion and characteristics (e.g., age, HIV status) of CC screened women who enroll in GC-CCP; and attend >3 sessions Benchmark of success will be at least 15% of CC screened women annually who receive over half of sessions, based on Diffusion of Innovation theory, which posits that ~15% of a target group should be exposed for optimal population diffusion of information	Data (enrollment, attendance dates) collected by clinic staff and added to medical chart (Implementation and Sustainment)
**Reach** (CC screened women and their alters)	b) Proportion and characteristics of research enrolled women who receive GC-CCP (i.e., index participants) who engage in advocacy	Survey data collected during RCT; intervention vs control comparison (Implementation)
	c) Proportion and characteristics of research enrolled alters who received advocacy from index participant	
	d) Proportion and characteristics of research enrolled alters who engage in advocacy with other alters	
**Effectiveness** (alters)	Proportion of previously unscreened alters who get screened for CC,	Med chart (intervention vs control group in RCT; Implementation)
**Adoption** (Clinic staff)	Clinic staff integrate GC-CCP (i.e., extent to which staff identify, train, and supervise peer facilitators, recruit clients, conduct sessions):	Twice yearly survey of clinic staff implementation activities (Sustainment)
	Proportion of staff who report recruiting clients for GC-CCP	Clinic staff supervisor fidelity forms (Sustainment)
	Extent to which staff supervisor carries out weekly supervision of peer facilitators conducting GC-CCP sessions	
**Implementation** (Fidelity)	Fidelity: Intervention facilitated as prescribed in manual (75% of session topics facilitated with fidelity across sessions)	Fidelity ratings (based on independent observations by 2 team members; Implementation & Sustainment)
**Implementation** (Perceptions of Implementation and Sustainment; EPIS domains)	a) Perceived acceptability (satisfaction with GC-CCP) (EPIS Innovation)	Semi-structured interviews with stakeholders (policy, clinic, and client levels); (Implementation & Sustainment)
	b) Appropriateness (perceived fit/compatibility of GC-CCP with current practices) (EPIS Innovation & Inner Context factors)	
	c) Feasibility (extent to which GC-CCP can be successfully conducted by clinics staff) (EPIS Innovation & Inner Context factors)	Brief surveys with health care providers, using adapted measures (Implementation & Sustainment)
	d) Other barriers to and facilitators of implementation	
**Implementation** (Adaptation)	Documentation of adaptations and recommendations needed to promote and sustain implementation	Semi-structured interviews (clinic staff) (Implementation & Sustainment)
**Implementation** (Cost)	Cost of implementation strategy	Clinic cost ledger (e.g., staff salaries, materials, space rental) (Sustainment)
**Maintenance** (assessed in “Sustainment” Phase only, last 2.5 years of data collection)	Implementation maintained over 2.5 years (i.e., GC-CCP conducted with at least 15% of women screened for CC; may be revised based on Implementation Phase evaluation)	Clinic records of GC-CCP implementation and client engagement (Sustainment)
	Supervision support for peer facilitators maintained over 2.5 years	Completed fidelity forms by clinic supervisor (Sustainment)
	Women receiving the intervention sustain advocacy 12-months post GC-CCP participation	Index client survey during RCT (Implementation & Sustainment)

*Semi-structured interviews with clinic staff*. We will assess implementation qualitatively with semi-structured interviews with clinic staff who are involved in CC screening and GC-CCP (e.g., clinic manager, GC-CCP peer facilitator, CC screener/treater). We will interview 20 staff (5 per clinic) at the beginning of Sustainment, followed by 15 and 30 months later (ideally, all with the same staff), to complement the quantitative outcomes in helping to understand the implementation process. Staff will be assured of confidentiality and that their responses will not be shared with others at the workplace. We will use the interview guide developed for the Preparation phase, revising questions as needed to ask about current practices and adding probes about specific barriers that emerged in prior interviews, to assess changes in barriers over time. We also will ask what resources are needed to sustain CC screening and GC-CCP.

*Client focus groups*. Using chart data on GC-CCP participation and CC screening referrals, a random sample of 10 clients per site who participate in GC-CCP, and a random sample of 10 CC screened alter clients per site who were referred by a peer, will be asked to participate in one of two focus groups per site. Selected women will be stratified by age (age 18–35 vs. 36+). Client participants will be referred by clinic staff to the study team for consent processes. Examples of questions to be asked of GC-CCP participants include: How easy or challenging was it for you to engage with the training sessions? How useful was the content of the training? How influential was the training on your engagement in advocacy with other women? Examples of questions for the alters include: How much, if at all, did the advocacy you receive address the questions and/or concerns you had about CC screening? How influential was the advocacy on your decision to get screened? How do you feel about advocating to other women?

*Survey data (clinic staff)*. To complement the qualitative data on implementation and sustainability factors, we will collect brief survey data from clinic staff once at the beginning and once at end of the 30-month Sustainment phase. These data will include adapted survey items [53] from three companion measures focused on the fit of GC-CCP with the clinic operations (e.g., “I welcome GC-CCP into the clinic,” “GC-CCP is a good fit with clinic practices,” and “It is possible to offer GC-CCP,” from 1 = completely disagree to 5 = completely agree). The questions will be tailored to the Ugandan clinic contexts during the Preparation phase of the project. In addition, to assess ***adoption***, clinic staff will be given a quarterly anonymous brief survey to assess if they helped to implement GC-CCP, and if so, the extent to which they performed key activities (e.g., identification and recruitment of CC screened clients to participate in GC-CCP; training or supervision of facilitators). A secure anonymous survey link will be emailed to providers, or they will be given a hard copy to complete and insert into an envelope. We will convene clinic providers and leadership quarterly to assess implementation challenges (re. CC screening and GC-CCP) that are raised in the surveys or during the meeting, and how to address them.

*Data analysis*. The qualitative data will be analyzed using methods similar to the Preparation phase. A longitudinal qualitative analysis will compare themes within and across timepoints to determine change in barriers over time (e.g., using summative analytic memos about themes at each timepoint) [54]. We will triangulate data collected from medical charts, semi-structured interviews and brief clinic surveys on CC screening uptake using a mixed methods approach [55].

### Trial coordination

The day-to-day study activities are coordinated directly by the Project Director, in tandem with the Study Coordinators, and all activities are overseen by the Senior Investigators. The Study Coordinators will recruit and track participants, as well as administer data collection tools. The Study Coordinators will also coordinate the logistics of planning the intervention sessions. The Project Director supervises and provides direct oversight of the Study Coordinators, whom she meets with weekly; she will also supervise the intervention facilitators and meet with them weekly in a separate meeting. The Senior Investigators provided oversight of the Project Director and Study Coordinators via twice-a-month meetings during which progress with study activities are reviewed and any challenges are addressed as a team.

### Community engagement

In both study settings (Kampala and rural area near Jinja) we will compose a Community Advisory Board comprised of key stakeholders (women in the community, health care providers, clinic administrators, district health officials). These boards will meet with the study team at key junctures of the study to provide input on the assessment instruments, intervention implementation, interpretation of results, and next steps for intervention dissemination, if it is shown to be cost-effective. Members will receive transport costs and refreshments at each meeting, which we expect to be held approximately twice a year.

### Ethics and dissemination

The study protocol has been approved by the institutional review boards (IRB) at Makerere University, College of Health Sciences, School of Public Health, and the RAND Corporation, as well as cleared by the Uganda National Council of Science and Technology. Any protocol modifications will be submitted to the IRBs for review, and participants will be informed if warranted. The procedures used in this study adhere to the tenets of the Declaration of Helsinki. Informed consent will be obtained from all individual participants included in the study.

To ensure and maintain the scientific integrity of this human subject research project, and to protect the safety of its research participants, we have a three member Data Safety Monitoring Board (DSMB) that will intermittently (at 6-month intervals) monitor study adverse event data. The DSMB will be provided with periodic reports which include subject enrollment, subject retention, reasons for dropping out, and a listing of all adverse events that are plausibly related to the intervention or study procedures. Adverse events that are considered directly related to the intervention or other aspect of study participation will be reported immediately to the DSMB, the IRBs, and NIH. After review of the periodic reports, the DSMB will make a recommendation regarding the continuation, modification, or termination of the study to the study senior investigators, who will make the final decision regarding continuation or termination. All communications from the DSMB will be shared with the IRBs and NIH. To protect confidentiality, all research data will be kept in locked file cabinets and/or secure password protected computers, and will be available only to members of the study team. Data will be identifiable only by study ID numbers. Personal information including participants’ name, address, and phone number will be stored separately from all research data. All data collected will be kept confidential and not shared with the client’s physician or other clinic staff, or any of their social network members whom they may recruit to participate.

As a first step for dissemination, reporting results will be documented on ClinicalTrials.gov in accordance with NIH requirements on dissemination of clinical trial results. Results submitted will occur no later than 12 months after the primary completion date. Findings produced by this investigation will be presented at international conferences and published in a timely fashion, ideally in the last year of the study period. All members of the study team will be eligible for authorship if they meet standard guidelines for contribution to the manuscript. All final peer-reviewed manuscripts that arise from this proposal will be submitted to the digital archive PubMed Central for open access. De-identified data, assessment and intervention materials, and analytic code will be made available upon request from external researchers and following review and approval of the study team.

## Discussion

Cervical cancer (CC) is the leading cause of cancer-related deaths among women in Uganda, yet very few women have ever been screened for CC. Furthermore, cancer treatment is scarcely available and too costly for most women, highlighting the importance of timely periodic screening to prevent onset of cancer. Drawing on theories of network diffusion and social influence, we developed the peer advocacy intervention *Game Changers for Cervical Cancer Prevention (GC-CCP)* to increase CC screening.

The hybrid type 1 RCT of GC-CCP described here has several innovations that could enable it to have a strong impact on CC prevention and control in Uganda. First, we are unaware of any other study that has used a network-driven approach and empowerment of CC screened peers as change agents to increase CC screening, in any setting, despite empirical evidence that peer support related factors facilitate CC screening uptake.

Second, the study will examine the potential for a multiplier effect of the advocacy training. The pilot of GC-CCP showed effects of increased CC advocacy among intervention recipients, as well as their social network members (alters) who were targeted with advocacy [30–32]. Diffusion of advocacy may heighten uptake of CC screening throughout networks and the larger community through increased CC knowledge, peer support, and stigma reduction. Unlike the pilot, the current project will assess the prevalence, quality and effects of advocacy conducted by first degree alters of the index participants, and the social network members of these alters (second degree alters), as well as index participants. We will explore how these intervention effects may attenuate the further the advocate is from direct exposure to the GC-CCP advocacy training.

Third, most hybrid designs rely on the study team to train and supervise intervention personnel, despite the need to study these implementation processes in a real-world context. We will start with the study team training facilitators to implement GC-CCP, but then transfer training and supervision duties to clinic staff who will be trained and mentored to take on this role during the sustainment phase of the study. This will enable us to identify barriers and facilitators to implementation and sustainability of GC-CCP across diverse clinic settings.

If shown to be an effective implementation strategy for promoting health screening and other health behaviors, this peer advocacy model could be applied to other disease contexts in both low-to-middle-income and high-resource settings.

### Trial status

Protocol version 1.0 (September 10, 2023); recruitment for RCT began August 20, 2024 and is expected to be completed in August 2025.

## Supporting information

S1 Checklist SPIRIT checklist. (DOCX)

S1 File Study protocol. (PDF)

S2 File PLOS’ questionnaire on inclusivity in global research. (DOCX)

## References

[1] Ferlay J, Ervik M, Lam F, Colombet M, Mery L, Piñeros M, et al. Global Cancer Observatory: Cancer Today. 2024. [cited 2024 November 25]. https://gco.iarc.who.int/media/globocan/factsheets/populations/900-world-fact-sheet.pdf.

[2] African Cancer Registry Network. Kampala Cancer Registry. 2018. [cited 2024, November 25]. https://www.afcrn.org/membership/81-kampala-uganda.

[3] Wabinga H, Parkin D, Nambooze S. Kampala Cancer Registry report for the period 2007–2009. Kampala, Uganda: Kampala Cancer Registry. 2012.

[4] Nakisige C, Schwartz M, Ndira AO. Cervical cancer screening and treatment in Uganda. Gynecologic Oncology Reports. 2017 Feb 3;20:37–40. doi: 10.1016/j.gore.2017.01.009 28275695 PMC5331149

[5] Nooh AM, Mohamed ME-S, El-Alfy Y. Visual Inspection of Cervix With Acetic Acid as a Screening Modality for Cervical Cancer. Journal of Lower Genital Tract Disease. October 2015;19(4). doi: 10.1097/LGT.0000000000000145 26247262

[6] World Health Organization. WHO guidelines for the use of thermal ablation for cervical pre-cancer lesions: executive summary. Geneva: 2019 9241550597. 31661202

[7] Ndejjo R, Mukama T, Musabyimana A, Musoke D. Uptake of Cervical Cancer Screening and Associated Factors among Women in Rural Uganda: A Cross Sectional Study. PLoS ONE. 2016 Feb 19;11(2). doi: 10.1371/journal.pone.0149696 26894270 PMC4760951

[8] Bruni L AG, Serrano B, Mena M, Collado JJ, Gómez D, Muñoz J, et al. Human Papillomavirus and Related Diseases in Uganda. Summary Report 10 March 2023. ICO/IARC Information Centre on HPV and Cancer (HPV Information Centre). 2023.

[9] Union for International Cancer Control (UICC). Cervical cancer elimination in Africa: where are we now and where do we need to be? 2022.

[10] Uganda Ministry of Health. Strategic Plan for Cervical Cancer Prevention and Control in Uganda 2010–2014. Kampala, UGANDA: Uganda Ministry of Health; 2010.

[11] Black E, Hyslop F, Richmond R. Barriers and facilitators to uptake of cervical cancer screening among women in Uganda: a systematic review. BMC Women’s Health. 2019-08-09;19(1). doi: 10.1186/s12905-019-0809-z 31399092 PMC6688246

[12] Teng FF, Mitchell SM, Sekikubo M, Biryabarema C, Byamugisha JK, Steinberg M, et al. Understanding the role of embarrassment in gynaecological screening: a qualitative study from the ASPIRE cervical cancer screening project in Uganda. BMJ Open. 2014;4(4). doi: 10.1136/bmjopen-2014-004783 24727360 PMC3987737

[13] Paul P, Winkler JL, Bartolini RM, Penny ME, Huong TT, Nga LT, et al. Screen-and-treat approach to cervical cancer prevention using visual inspection with acetic acid and cryotherapy: experiences, perceptions, and beliefs from demonstration projects in Peru, Uganda, and Vietnam—PubMed. The oncologist. 2013;18(12). doi: 10.1634/theoncologist.2013-0253 24217554 PMC3868422

[14] Ndejjo R, Mukama T, Kiguli J, Musoke D. Knowledge, facilitators and barriers to cervical cancer screening among women in Uganda: a qualitative study. BMJ Open. 2017;7(6). doi: 10.1136/bmjopen-2017-016282 28606908 PMC5541520

[15] Medley A, Kennedy C, O’Reilly K, Sweat M. Effectiveness of Peer Education Interventions for HIV Prevention in Developing Countries: A Systematic Review and Meta-Analysis. 2009;21(3). doi: 10.1521/aeap.2009.21.3.181 19519235 PMC3927325

[16] Maiorana A, Kegeles S, Fernandez P, Salazar X, Cáceres C, Sandoval C, et al. Implementation and evaluation of an HIV/STD intervention in Peru. Evaluation and Program Planning. 2007;30(1):82–93. doi: 10.1016/j.evalprogplan.2006.10.004 17689315 PMC2739095

[17] Rogers EM. Diffusion of innovations. 5th ed. New York: Free Press; 2003.

[18] Broadhead RS, Heckathorn DD, Weakliem DL, Anthony DL, Madray H, Mills RJ, et al. Harnessing peer networks as an instrument for AIDS prevention: results from a peer-driven intervention. Public Health Reports. 1998;113 Suppl 1(Suppl 1):42–57. . 9722809 PMC1307726

[19] Kelly JA. Popular opinion leaders and HIV prevention peer education: resolving discrepant findings, and implications for the development of effective community programmes. AIDS Care. 2004-2-1;16(2). doi: 10.1080/09540120410001640986 14676020

[20] Bell DC, Montoya ID, Atkinson JS, Yang S-J. Social Networks and Forecasting the Spread of HIV Infection. JAIDS Journal of Acquired Immune Deficiency Syndromes. October 1, 2002;31(2). doi: 10.1097/01.QAI.0000026510.98625.9A 12394801

[21] Latkin CA, Sherman S, Knowlton A. HIV prevention among drug users: Outcome of a network-oriented peer outreach intervention. Health Psychology. 2003;22(4):332–9. 2003-05896-003. doi: 10.1037/0278-6133.22.4.332 12940388

[22] Sikkema KJ, Kelly JA, Winett RA, Solomon LJ, Cargill VA, Roffman RA, et al. Outcomes of a randomized community-level HIV prevention intervention for women living in 18 low-income housing developments. American Journal of Public Health. 2000 Jan;90(1). doi: 10.2105/ajph.90.1.57 10630138 PMC1446110

[23] Friedman Samuel R., Maslow Carey, Bolyard Melissa, Sandoval Milagros, Mateu-Gelabert Pedro, Neaigus A. Urging Others to be Healthy: “Intravention” by Injection Drug Users as a Community Prevention Goal. 2005-06-01;16(3). doi: 10.1521/aeap.16.3.250.35439 15237054

[24] Li M, Nyabigambo A, Navvuga P, Nuwamanya E, Nuwasiima A, Kaganda P, et al. Acceptability of cervical cancer screening using visual inspection among women attending a childhood immunization clinic in Uganda. Papillomavirus Research. 2017;4. doi: 10.1016/j.pvr.2017.06.004 29179864 PMC5883247

[25] Mutyaba T, Mirembe F, Sandin S, Weiderpass E. Male partner involvement in reducing loss to follow-up after cervical cancer screening in Uganda. International Journal of Gynecology & Obstetrics. 2009/11/01;107(2). doi: 10.1016/j.ijgo.2009.07.019 19716557

[26] Roura M, Urassa M, Busza J, Mbata D, Wringe A, Zaba B. Scaling up stigma? The effects of antiretroviral roll-out on stigma and HIV testing. Early evidence from rural Tanzania. Sexually Transmitted Infections. 2009-08-01;85(4). doi: 10.1136/sti.2008.033183 19036776 PMC2708343

[27] Uganda Ministry of Health; ORC Macro; Centers for Disease Control and Prevention (U.S.) Uganda HIV/AIDS Sero-behavioural Survey: 2004–2005. Kampala, Uganda; Calverton, MD: Ministry of Health, 2006.

[28] Nam SL, Fielding K, Avalos A, Dickinson D, Gaolathe T, Geissler PW. The relationship of acceptance or denial of HIV-status to antiretroviral adherence among adult HIV patients in urban Botswana. Social Science & Medicine. 2008/07/01;67(2). doi: 10.1016/j.socscimed.2008.03.042 18455285

[29] Wagner G, Ryan G, Huynh A, Kityo C, Mugyenyi P. A Qualitative Analysis of the Economic Impact of HIV and Antiretroviral Therapy on Individuals and Households in Uganda. 2009-09-09;23(9). doi: 10.1089/apc.2009.0028 19663715

[30] Wagner GJ, Matovu JKB, Juncker M, Namisango E, Bouskill K, Nakami S, et al. Effects of a peer advocacy intervention on cervical cancer screening among social network members: results of a randomized controlled trial in Uganda. Journal of Behavioral Medicine. 2023-09-13;46(6). doi: 10.1007/s10865-023-00418-6 37702912 PMC10577098

[31] Matovu JK, Wagner GJ, Juncker M, Namisango E, Bouskill K, Nakami S, et al. Mediators and moderators of the effect of the game changers for cervical cancer prevention intervention on cervical cancer screening among previously unscreened social network members in Uganda. BMC Cancer. 2023 May 11;23(1). doi: 10.1186/s12885-023-10924-0 37170099 PMC10173559

[32] Wagner GJ, Matovu JKB, Juncker M, Namisango E, Beyeza-Kashesya J, Wanyenze RK. Knowledge Mediates the Effects of Game Changers for Cervical Cancer Prevention (GC-CCP) Intervention on Increased VIA Screening Advocacy in Uganda. Cancer Prevention Research. 2023/12/01;16(12). doi: 10.1158/1940-6207.CAPR-23-0262 37768937 PMC10843060

[33] Aarons GA, Hurlburt M, Horwitz SM, Aarons GA, Hurlburt M, Horwitz SM. Advancing a Conceptual Model of Evidence-Based Practice Implementation in Public Service Sectors. Administration and Policy in Mental Health and Mental Health Services Research. 2010-12-14;38(1). doi: 10.1007/s10488-010-0327-7 21197565 PMC3025110

[34] Moullin JC, Dickson KS, Stadnick NA, Rabin B, Aarons GA. Systematic review of the Exploration, Preparation, Implementation, Sustainment (EPIS) framework. Implementation Science. 2019;14(1). doi: 10.1186/s13012-018-0842-6 30611302 PMC6321673

[35] Glasgow RE, Vogt TM, Boles SM. Evaluating the public health impact of health promotion interventions: the RE-AIM framework. American Journal of Public Health. 1999 Sep;89(9). doi: 10.2105/ajph.89.9.1322 10474547 PMC1508772

[36] Chan A-W, Tetzlaff JM, Gøtzsche PC, Altman DG, Mann H, Berlin JA, et al. SPIRIT 2013 explanation and elaboration: guidance for protocols of clinical trials. BMJ. 2013-01-09;346(jan08 15). doi: 10.1136/bmj.e7586 23303884 PMC3541470

[37] Uganda Ministry of Health. Annual Health Sector Performance Report 2020/2021. Kampala: Uganda Ministry of Health; 2021.

[38] Proctor E, Silmere H, Raghavan R, Hovmand P, Aarons G, Bunger A, et al. Outcomes for Implementation Research: Conceptual Distinctions, Measurement Challenges, and Research Agenda. Administration and Policy in Mental Health and Mental Health Services Research. 2010-10-19;38(2). doi: 10.1007/s10488-010-0319-7 20957426 PMC3068522

[39] Bernard HR. Research Methods in Anthropology: Qualitative and Quantitative Approaches. 5th ed. CA: AltaMira Press; 2006. xvi–xvi p.

[40] Jehn KA, Doucet L, Karen A. Jehn LD. Developing Categories for Interview Data: Consequences of Different Coding and Analysis Strategies in Understanding Text: Part 2. CAM Journal. 1997-02-01;9(1). doi: 10.1177/1525822X970090010101

[41] Huberman AM, Miles MB. Data Management and Analysis Methods. In: Denzin N LY, editor. Handbook of Qualitative Research. 2nd ed. Thousand Oaks: Sage Publications; 1994. p. 428–44.

[42] Cohen J. A Coefficient of Agreement for Nominal Scales. Educational and Psychological Measurement. 1960;20(1). doi: 10.1177/001316446002000104

[43] Deming WE. Out of the crisis. Cambridge, Massachusetts: The MIT Press; 2018.

[44] Berwick DM. Developing and Testing Changes in Delivery of Care. Annals of Internal Medicine. 2000-08-15;128(8). doi: 10.7326/0003-4819-128-8-199804150-00009 9537939

[45] Chinman M, Hunter SB, Ebener P. Employing continuous quality improvement in community- based substance abuse programs. International journal of health care quality assurance. 2012;25(7):604–17. doi: 10.1108/09526861211261208 23276056 PMC5646166

[46] Hunter S, Ebener PA, Chinman M, Ober AJ, Huang CY, Health R, et al. Promoting success: a Getting To Outcomes^®^ guide to implementing continuous quality improvement for community service organizations. 3 (Publicly Releasable) ed. Santa Monica, CA: RAND; 2015.

[47] Damschroder LJ, Aron DC, Keith RE, Kirsh SR, Alexander JA, Lowery JC. Fostering implementation of health services research findings into practice: a consolidated framework for advancing implementation science. Implementation Science. 2009 Aug 7;4(1). doi: 10.1186/1748-5908-4-50 19664226 PMC2736161

[48] Curran GM, Bauer M, Mittman B, Pyne JM, Stetler C. Effectiveness-implementation Hybrid Designs: Combining Elements of Clinical Effectiveness and Implementation Research to Enhance Public Health Impact. Medical Care. March 2012;50(3). doi: 10.1097/MLR.0b013e3182408812 22310560 PMC3731143

[49] Gold Marthe R S JE, Russell Louise B, Weinstein Milton C, editor. Cost-Effectiveness in Health and Medicine. Online ed. New York, NY: Oxford University Press; 1996.

[50] Campbell MK, Torgerson DJ. Bootstrapping: estimating confidence intervals for cost-effectiveness ratios. QJM: An International Journal of Medicine. 1999/03/01;92(3). doi: 10.1093/qjmed/92.3.177 10326078

[51] Gorsky RD. A Method to Measure the Costs of Counseling for HIV Prevention. Public Health Reports. 1996;111:115–22. 8862166 PMC1382052

[52] Creese A, Parker D. Cost analysis in primary health care: a training manual for programme managers: World Health Organization; 1994.

[53] Weiner BJ, Lewis CC, Stanick C, Powell BJ, Dorsey CN, Clary AS, et al. Psychometric assessment of three newly developed implementation outcome measures. Implementation Science: IS. 2017 Aug 29;12(1). doi: 10.1186/s13012-017-0635-3 28851459 PMC5576104

[54] Barrington C, Rosenberg A, Kerrigan D, Blankenship KM, Barrington C, Rosenberg A, et al. Probing the Processes: Longitudinal Qualitative Research on Social Determinants of HIV. AIDS and Behavior. 2021-03-27;25(2). doi: 10.1007/s10461-021-03240-w 33772696 PMC8473579

[55] Palinkas LA, Aarons GA, Horwitz S, Chamberlain P, Hurlburt M, Landsverk J, et al. Mixed Method Designs in Implementation Research. Administration and Policy in Mental Health and Mental Health Services Research. 2010-10-22;38(1). doi: 10.1007/s10488-010-0314-z 20967495 PMC3025112

