# Supplementary material for: Study protocol for a hybrid implementation-effectiveness trial of Game Changers for Cervical Cancer Prevention in Uganda
Source: PLoS One. 2025 Jan 24;20(1):e0317491. doi: 10.1371/journal.pone.0317491 (PMC11760014; doi:10.1371/journal.pone.0317491)
Supplement: S1 Checklist — (DOCX) [file pone.0317491.s001.docx]

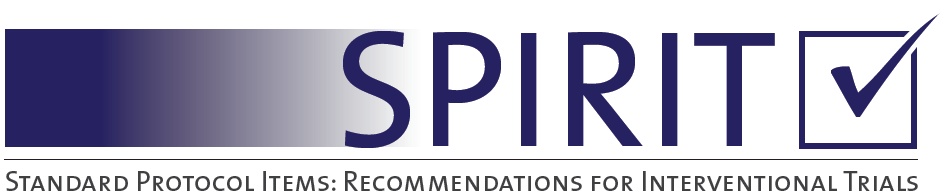


SPIRIT 2013 Checklist: Recommended items to address in a clinical trial protocol and related documents*

**Section/item** **Item** **Description** **No**

**Addressed** **on** **page** **number**

**Administrative** **information**

Title

Trial registration

1 Descriptive title identifying the study design, population, interventions, and, if applicable, trial acronym

2a Trial identifier and registry name. If not yet registered, name of intended registry

______1_______

______2______

2b All items from the World Health Organization Trial Registration Data Set ___refer to registered trial (2a)__________

Protocol version

Funding

Roles and

responsibilities

3 Date and version identifier

4 Sources and types of financial, material, and other support

5a Names, affiliations, and roles of protocol contributors

5b Name and contact information for the trial sponsor

______26_______

_______27______

_______27______

_______27______

5c Role of study sponsor and funders, if any, in study design; collection, management, analysis, and interpretation of data; writing of the report; and the decision to submit the report for publication, including whether they will have ultimate authority over any of these activities

5d Composition, roles, and responsibilities of the coordinating centre, steering committee, endpoint adjudication committee, data management team, and other individuals or groups overseeing the trial, if

applicable (see Item 21a for data monitoring committee)

_____27________

____23________

1

**Introduction**

Background and

rationale

6a Description of research question and justification for undertaking the trial, including summary of relevant ____4-5________

studies (published and unpublished) examining benefits and harms for each intervention

6b Explanation for choice of comparators ____11-12_____

Objectives

Trial design

7 Specific objectives or hypotheses

8 Description of trial design including type of trial (eg, parallel group, crossover, factorial, single group),

allocation ratio, and framework (eg, superiority, equivalence, noninferiority, exploratory)

_______6______

6

_____________

**Methods:** **Participants,** **interventions,** **and** **outcomes**

Study setting

Eligibility criteria

Interventions

9 Description of study settings (eg, community clinic, academic hospital) and list of countries where data will be collected. Reference to where list of study sites can be obtained

10 Inclusion and exclusion criteria for participants. If applicable, eligibility criteria for study centres and individuals who will perform the interventions (eg, surgeons, psychotherapists)

11a Interventions for each group with sufficient detail to allow replication, including how and when they will be

administered

________7_____

_________13____

_______11______

11b Criteria for discontinuing or modifying allocated interventions for a given trial participant (eg, drug dose change in response to harms, participant request, or improving/worsening disease)

11c Strategies to improve adherence to intervention protocols, and any procedures for monitoring adherence (eg, drug tablet return, laboratory tests)

11d Relevant concomitant care and interventions that are permitted or prohibited during the trial

_______12_____

_________12-13_

_______12___

Outcomes 12 Primary, secondary, and other outcomes, including the specific measurement variable (eg, systolic blood

pressure), analysis metric (eg, change from baseline, final value, time to event), method of aggregation (eg, ____15-16____ median, proportion), and time point for each outcome. Explanation of the clinical relevance of chosen

efficacy and harm outcomes is strongly recommended

Participant timeline 13 Time schedule of enrolment, interventions (including any run-ins and washouts), assessments, and visits for ___15________ participants. A schematic diagram is highly recommended (see Figure)

2

Sample size

Recruitment

14 Estimated number of participants needed to achieve study objectives and how it was determined, including clinical and statistical assumptions supporting any sample size calculations

15 Strategies for achieving adequate participant enrolment to reach target sample size

___12-13_______

_____13-14____

**Methods:** **Assignment** **of** **interventions** **(for** **controlled** **trials)**

Allocation:

Sequence 16a generation

Allocation 16b concealment mechanism

Implementation 16c

Blinding (masking) 17a

Method of generating the allocation sequence (eg, computer-generated random numbers), and list of any factors for stratification. To reduce predictability of a random sequence, details of any planned restriction (eg, blocking) should be provided in a separate document that is unavailable to those who enrol participants or assign interventions

Mechanism of implementing the allocation sequence (eg, central telephone; sequentially numbered, opaque, sealed envelopes), describing any steps to conceal the sequence until interventions are assigned

Who will generate the allocation sequence, who will enrol participants, and who will assign participants to interventions

Who will be blinded after assignment to interventions (eg, trial participants, care providers, outcome

assessors, data analysts), and how

_____15________

_____15________

______15_______

______15_______

17b If blinded, circumstances under which unblinding is permissible, and procedure for revealing a participant’s ____open label design_________ allocated intervention during the trial

**Methods:** **Data** **collection,** **management,** **and** **analysis**

Data collection

methods

18a Plans for assessment and collection of outcome, baseline, and other trial data, including any related _____15______ processes to promote data quality (eg, duplicate measurements, training of assessors) and a description of

study instruments (eg, questionnaires, laboratory tests) along with their reliability and validity, if known.

Reference to where data collection forms can be found, if not in the protocol

18b Plans to promote participant retention and complete follow-up, including list of any outcome data to be ____15_______ collected for participants who discontinue or deviate from intervention protocols

3

Data management 19

Statistical methods 20a

Plans for data entry, coding, security, and storage, including any related processes to promote data quality (eg, double data entry; range checks for data values). Reference to where details of data management procedures can be found, if not in the protocol

Statistical methods for analysing primary and secondary outcomes. Reference to where other details of the

statistical analysis plan can be found, if not in the protocol

__24_________

____17_______

20b Methods for any additional analyses (eg, subgroup and adjusted analyses)

20c Definition of analysis population relating to protocol non-adherence (eg, as randomised analysis), and any

statistical methods to handle missing data (eg, multiple imputation)

______17_____

______17_____

**Methods:** **Monitoring**

Data monitoring 21a Composition of data monitoring committee (DMC); summary of its role and reporting structure; statement of ____24_______ whether it is independent from the sponsor and competing interests; and reference to where further details

about its charter can be found, if not in the protocol. Alternatively, an explanation of why a DMC is not needed

21b Description of any interim analyses and stopping guidelines, including who will have access to these interim ________24___ results and make the final decision to terminate the trial

Harms

Auditing

22 Plans for collecting, assessing, reporting, and managing solicited and spontaneously reported adverse events and other unintended effects of trial interventions or trial conduct

23 Frequency and procedures for auditing trial conduct, if any, and whether the process will be independent

from investigators and the sponsor

_______24____

_______24____

**Ethics** **and** **dissemination**

Research ethics approval

Protocol

amendments

24 Plans for seeking research ethics committee/institutional review board (REC/IRB) approval regulators

25 Plans for communicating important protocol modifications (eg, changes to eligibility criteria, outcomes, analyses) to relevant parties (eg, investigators, REC/IRBs, trial participants, trial registries, journals,

___24________

______24_____

4

Consent or assent 26a Who will obtain informed consent or assent from potential trial participants or authorised surrogates, and ___14________ how (see Item 32)

26b Additional consent provisions for collection and use of participant data and biological specimens in ancillary __N/A_______ studies, if applicable

Confidentiality 27 How personal information about potential and enrolled participants will be collected, shared, and maintained _______24____ in order to protect confidentiality before, during, and after the trial

Declaration of 28 interests

Access to data 29

Ancillary and post- 30 trial care

Dissemination policy 31a

Financial and other competing interests for principal investigators for the overall trial and each study site

Statement of who will have access to the final trial dataset, and disclosure of contractual agreements that limit such access for investigators

Provisions, if any, for ancillary and post-trial care, and for compensation to those who suffer harm from trial participation

Plans for investigators and sponsor to communicate trial results to participants, healthcare professionals, the public, and other relevant groups (eg, via publication, reporting in results databases, or other data

sharing arrangements), including any publication restrictions

______27_____

_______27____

_______14____

______25_____

31b Authorship eligibility guidelines and any intended use of professional writers

31c Plans, if any, for granting public access to the full protocol, participant-level dataset, and statistical code

_______25____

________25___

**Appendices**

Informed consent 32 materials

Biological 33

specimens

Model consent form and other related documentation given to participants and authorised surrogates

Plans for collection, laboratory evaluation, and storage of biological specimens for genetic or molecular

analysis in the current trial and for future use in ancillary studies, if applicable

_ __

N/A_________

*It is strongly recommended that this checklist be read in conjunction with the SPIRIT 2013 Explanation & Elaboration for important clarification on the items. Amendments to the protocol should be tracked and dated. The SPIRIT checklist is copyrighted by the SPIRIT Group under the Creative Commons [“Attribution-NonCommercial-NoDerivs 3.0 Unported”](http://www.creativecommons.org/licenses/by-nc-nd/3.0/) license.

5
