# Supplementary material for: Study protocol for a hybrid implementation-effectiveness trial of Game Changers for Cervical Cancer Prevention in Uganda
Source: PLoS One. 2025 Jan 24;20(1):e0317491. doi: 10.1371/journal.pone.0317491 (PMC11760014; doi:10.1371/journal.pone.0317491)
Supplement: S1 File — (PDF) [file pone.0317491.s002.pdf]

RESEARCH PROPOSAL

A Hybrid Implementation-Effectiveness Trial of Game Changers for Cervical Cancer Prevention (GC-CCP) in Uganda

FUNDING AGENCY

National Institutes of Health, USA

Submitted to

Makerere University School of Public Health
Higher Degrees Research and Ethics Committee
Kampala, Uganda

April 14, 2023, version 2, revised 19th May 2024

Investigators

1. Glenn Wagner; RAND Corporation (PI-Overall Lead PI for the Grant)
2. Laura Bogart, RAND Corporation (Co-Principal Investigator)
3. Rhoda Wanyenze, Makerere University, School of Public Health (PI—Ugandan PI)
4. Jolly Beyeza-Kashesya; Mulago Hospital (Co-Investigator)
5. Joseph Matovu, Makerere University, School of Public Health (Co-Investigator)
6. Sylvia Nakami, Rays of Hope Hospice Jinja (Co-Investigator)
7. Eve Namisango, African Palliative Care Association (Co-Investigator)
8. Harold Green, Indiana University Bloomington (Co-Investigator)
9. Allison Ober RAND Corporation (Co-Investigator)

TABLE OF CONTENTS

TABLE OF CONTENTS 2
ABBREVIATIONS AND ACRONYMS 3
A. SPECIFIC AIMS 4
B. SIGNIFICANCE 5
C. INNOVATION 7
D. APPROACH 7
E. HUMAN SUBJECTS PROTECTION 17
F. DATA SAFETY AND MONITORING PLAN 18
G. STUDY TIMELINES 24
References 25

ABBREVIATIONS AND ACRONYMS

AES	Advanced Encryption Standard
CC	Cervical Cancer
EBP	Evidence-based practice
GC-CCP	*Game Changers for Cervical Cancer Prevention*
HIV	Human Immunodeficiency Virus
HPV	Human Papilloma Virus
ICC	Intra-Cluster Correlation
IS	Implementation science
IRB	Institutional Review Board
LMIC	Low and Middle Income Countries
MakSPH	Makerere University School of Public Health
MoH	Ministry Health
NIH	National Institutes Of Health
PDCA	Plan-Do-Check-Act
RCT	Randomized controlled trial
STI	Sexually Transmitted Infections
TDABC	Time-driven activity based costing
UCI	Uganda Cancer Institute
VIA	Visual Inspection with Acetic Acid

A. SPECIFIC AIMS

Cervical cancer (CC) is the leading cause of cancer-related deaths among women in Uganda, which has one of the world's highest incidence rates. Radiation therapy for advanced disease is scarcely available and too costly for most women, highlighting the importance of timely periodic screening and treatment to prevent onset of cancer. Evidence-based visual inspection with acetic acid (VIA) CC screening and thermal therapy for pre-cancerous lesions are free or low cost, yet ~ 5% of Ugandan women have ever screened for CC, and ~80% have advanced cancer when initiating care. Government efforts are ramping up availability of CC screening, but uptake remains very low. Innovative, sustainable implementation strategies to increase demand for CC screening are badly needed.

Drawing on theories of network diffusion and social influence, and known patient-level, non-structural barriers (poor CC knowledge; stigma and misconceptions) and facilitators of CC screening (peer support and knowing someone who has screened), we developed the peer advocacy intervention *Game Changers for Cervical Cancer Prevention* (GC-CCP; R21TW011728) as a patient-level implementation strategy to increase CC screening. GC-CCP empowers women who have recently screened for CC to encourage screening among women in their social networks by disseminating CC knowledge, dispelling myths and fears and reducing CC stigma, and facilitating motivation for CC screening. In a pilot randomized controlled trial (RCT) of the peer-led, 7-session group intervention, 40 women who had screened for CC (index participants) were assigned to the intervention (n=20) or wait-list control (n=20), from whom 103 of their social network members (referred to as "alters") who had never screened for CC were enrolled; at month-6, 64% of intervention alters had been screened for CC, compared to just 16% of control alters [OR (95% CI)=12.13 (4.07, 36.16)]. Also, not only did GC-CCP significantly increase CC prevention advocacy among intervention index participants, alters of those in the intervention group (who had no exposure to the intervention, other than indirectly through advocacy from index participants) also reported increased engagement in CC prevention advocacy, compared to controls. The promising results of this pilot, and the strong track record of successful collaboration by our multidisciplinary study team (experts related to CC in the local context, implementation science (IS), and development and evaluation of the network-based peer advocacy intervention), positions this application to make a strong impact on CC prevention and control in Uganda, which could be generalizable to other LMIC settings.

In response to NOT-CA-22-038, we propose a hybrid type 1 RCT of GC-CCP, a clinical intervention that also meets established criteria for a patient-level implementation strategy, for increasing uptake of evidence-based VIA screening. The study design is guided by the Exploration, Preparation, Implementation and Sustainment (EPIS) IS framework. <u>Exploration</u>: This phase consisted of the R21-funded pilot of GC-CCP and selection of study sites (two public, two private, one each in an urban and rural location). <u>Preparation</u>: In year 1, we will identify and remediate basic clinic- and provider-level barriers to ensure good access to and delivery of CC screening services, including provider training on the need to recommend CC screening to clients and necessary medical equipment for screening. <u>Implementation</u>: At each site, 40 women screened for CC will enroll in the RCT as index participants (total=160 index) and be randomized to the intervention (in two groups of 10) or wait-list control. Each index will recruit up to three (1st degree) alters (total= ~440 alters) at baseline who have not screened for CC; these index and alter participants will be followed up at months 6 and 12. At month 6, these alters will each recruit up to two (2nd degree) alters (total= ~800 alters) for a single phone interview. <u>Sustainment</u>: After the enrolled index participants receive the intervention, each clinic will continue to implement GC-CCP (with clients not enrolled in the RCT) on a quarterly basis for an additional 2.5 years, with training and supervision tasks transferred to clinic staff. Using the RE-AIM framework, we will evaluate engagement in the group intervention and CC advocacy (reach), alter CC screening (effectiveness), adoption into clinic operations, implementation outcomes (acceptability, feasibility, fidelity), and maintenance over time.
The specific aims are to:

1. Conduct a multisite RCT of the GC-CCP network-based advocacy strategy to evaluate effects on CC screening uptake among unscreened alters across urban/rural and public/private clinics.
2. Use a mixed methods approach (semi-structured interviews and administrative clinic data) to examine clinic-, provider-, and client-level barriers and facilitators of GC-CCP Implementation and Sustainment.
3. Examine mediators and moderators (among index, alter and network characteristics) of intervention effects on (a) alter CC screening; and (b) engagement in CC prevention advocacy of index and alter (1st and 2nd degree) to better understand its multiplier effect on diffusion of advocacy throughout a network.
4. Evaluate the cost-effectiveness of Implementing GC-CCP to increase CC screening and low cost, early-stage treatment, and prevent advanced disease, compared to enhanced usual care.

B. SIGNIFICANCE

Cervical cancer (CC) is the leading cause of cancer-related death among Uganda women, yet rates of CC screening are very low. CC accounts for ~25% of all cancer deaths in Ugandan women.[1-4] 80% of women presenting for care have advanced disease,[5] for whom radiotherapy is usually prescribed but is only available at the Uganda Cancer Institute (UCI) in Kampala and thus too far and too costly for most women. This highlights the critical need for timely, periodic screening with visual inspection of cervix with acetic acid (VIA), and early-stage treatment (thermal therapy for pre-cancerous lesions) when warranted, both of which are evidence-based standard of care,[6,7] as well as free or low cost in Uganda. Yet the lifetime screening rate for CC in Uganda is ~5%, despite an HPV prevalence of ~34%,[5,8,9] recommendations for CC screening every 3 years, and the WHO Global Strategy to accelerate elimination of CC striving for a 70% rate of CC screening.[10] CC screening access is generally limited to national and regional referral hospitals, private hospitals in Kampala and larger towns, and a few health centers in smaller towns. However, the Ministry of Health (MoH) has a current initiative to expand access across the country, making this an optimal time to increase demand for screening.

Structural and non-structural barriers to CC screening in Uganda have been identified. Structural barriers include limited access (screening is not available in all districts), cost of transport, and lack of active client education from providers,[11-12] and non-structural barriers such as lack of awareness and misinformation, embarrassment with the procedure (especially if the provider is male), fear of results, and stigma due to CC stemming from an STI and symptoms of advanced disease (fistulas, incontinence, heavy bleeding, offensive discharge) that lead women to be shunned and isolated.[12-16] Facilitators to screening include outreach, support and encouragement from others, and knowing someone who has been screened or diagnosed.[12,14,15] Despite these peer support related facilitators, *we are not aware of interventions that leverage and diffuse information through social networks to increase CC screening.* If the structural barriers can be minimized in the Preparation phase of the proposed study (e.g., provider sensitization to increase education of clients; ensuring access to female screeners), a network-based peer advocacy strategy holds great promise for addressing the non-structural barriers (e.g., dissemination of knowledge, peer support, stigma reduction) that have been identified.

Network-based peer advocacy has merit for increasing CC screening. Network-based interventions, such as the Peer Change Agent[17] and Popular Opinion Leader[18] models, draw on network diffusion theory[19] and principles of social identity and social influence[20] to posit that behavior change can be initiated by a few and diffused to others through modeling, advocacy, and shifts in norms.[21,22] Intervention effects on increased advocacy,[23] and improved health behavior of the recipients of advocacy have been observed,[24-25] but mostly in the context of HIV risk and drug use. In the context of CC, peer education has been evaluated in healthcare workers,[26] male partners,[27] and women at risk,[15,16] but not through clients' social networks. We developed the *Game Changers* model (GC-HIV) to train people living with HIV in Uganda to advocate for HIV protective behaviors within their social networks; we found that intervention recipients reported lower internal stigma, and increased HIV disclosure and advocacy, and their social network members reported increased HIV testing and condom use.[28] We then adapted and piloted the intervention model to CC screening promotion (GC-CCP), and found that the intervention had a strong effect on CC screening uptake among social network members (see D.1). Furthermore, not only did we find increased CC prevention advocacy among intervention recipients, but also the social network members whom they targeted with advocacy, suggesting a diffusion of advocacy effect within the network. Other network research shows changes in knowledge and behavior can have second and third degree effects within social networks,[29,30] further supporting the need to explore this effect in the proposed study. The diffusion of CC knowledge and CC screening practices throughout social networks, and thus whole communities, has the potential for making a tremendous impact on CC prevention and control.

Empowering women screened for CC to act as change agents for CC prevention requires advocacy training. GC-CCP targets both the advocates and their social network members by promoting processes (stigma reduction, sharing of CC screening experience, management of CC risk, CC knowledge, and advocacy skills) needed to support behavior change. *Effective advocacy first requires coping with fears and internal stigma*.[31,32] Coming to terms with one's CC diagnosis or risk (reflected in pre-cancerous or cancerous lesions, if present) helps self-acceptance, and is related to received stigma, discrimination and support.[33,34] Self-acceptance facilitates *sharing one's CC screening experience*, which enhances the credibility of one's advocacy for others to get screened. However, disclosure of CC risk can increase support as well as lead to rejection or ridicule, and thus disclosure decision-making skills are important. For effective advocacy, women must *model the behaviors they encourage others to adopt, by actively managing CC risk*, including recognizing signs of CC risk and seeking health services when needed, as well as timely periodic screening. *Learning communication skills for who, when and how to engage in advocacy* are of course key to effective advocacy.

These processes make up the framework (Fig. 1) of GC-CCP. It draws on evidence-based network-driven interventions[35-38] and the social influence theories noted above. To the extent that CC prevention advocacy may diffuse throughout a network to reach women two or three degrees of separation from direct recipients of the intervention, our research will examine how prevalence, quality and impact of advocacy may be sustained during the diffusion process. *If GC-CCP fosters collective use of advocacy among networks of women, it could reduce stigma in the community, build peer support and solidarity among women at risk, and greatly impact CC prevention and control.*

Figure 1. Conceptual framework for promotion of cervical cancer (CC) prevention advocacy among CC screened women to affect CC screening among social

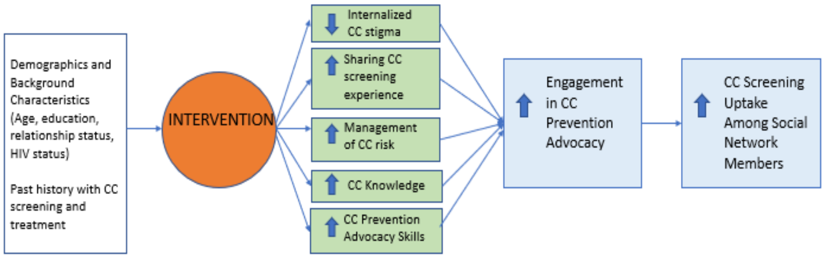

The Exploration, Preparation, Implementation and Sustainability (EPIS) framework guides our work.

Fig 2.EPIS Framework Guides CC Screening and GC-CCP-CCP Implementation and Sustainment

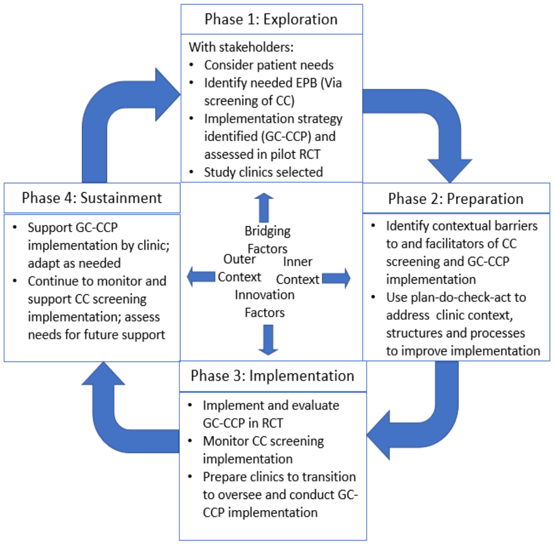

The pilot of GC-CCP (see D.1) provides preliminary evidence of its efficacy for increasing CC screening, but it is important to further establish its effectiveness, and assess how it can be implemented and sustained in diverse settings. The EPIS framework[39,40] will help us to understand the factors needed to ensure adequate CC screening [the evidence-based practice (EBP)] across clinics prior to our RCT, as well as a sustainable implementation of GC-CCP (the implementation strategy) as it is integrated into clinic operations. EPIS has 4 implementation phases and identifies factors, and the interplay among them, that influence implementation and sustainability. Factors within ("Inner Context") and outside the clinic ("Outer Context"), government, community, and research partnerships ("Bridging Factors"), characteristics of the EBP and GC-CCP (implementation strategy) and their effectiveness, ease of use, and organizational fit ("Innovation Factors"), can all affect implementation. Fig 2 shows how EPIS is applied to our study. Some factors were assessed in our pilot work (Exploration phase); the Preparation phase in the proposed study will further assess these factors.

<u>"Inner setting" factors</u> we will assess and address in the Preparation and Sustainment phases include support from leadership, staff capacity (knowledge/skills for performing VIA screening and thermal therapy, and training and supervising peer facilitators for GC-CCP) and motivation to provide services (even if overburdened), and available resources (equipment/supplies, trained labor, small incentives for GC-CCP peer facilitators) to sustain VIA, thermal therapy and GC-CCP. The leadership and staff of our study clinics have pledged strong support for integrating GC-CCP into their system of care, but we will assess this formally during the study.

<u>"Outer setting" factors</u> that can affect CC screening and GC-CCP implementation and sustainment are MoH policies that recommend but provide limited funding for training and equipment for CC screening and treatment; client factors; and external funding (e.g., USAID, PEPFAR) that prioritize screening of HIV+ women, inadvertently undercutting provider incentives to screen all women. We will revisit these policies prior to and after implementation to determine whether they have changed to improve access to CC screening. The MoH is a partner on the study team and can help address structural issues at a policy level.

<u>Client factors</u> for CC screening/thermal therapy, and participation in GC-CCP and CC advocacy, were noted in our Exploratory work and will be measured in the study: knowledge/misconceptions of CC disease, screening and treatment (e.g., VIA and/or thermal therapy will damage the uterus, or impede childbearing), CC stigma, partner support, and screening result (screening positive may motivate participation and advocacy).[12-16]

<u>"Innovation" factors</u> pertain to both the EBP (VIA screening and thermal therapy) and GC-CCP. For the former, factors include the cultural sensitivity of inspecting women's genitals (especially if the provider is male), and the 6-week interruption of sexual intercourse necessitated by thermal therapy (which can create conflict with male partners). For GC-CCP, factors include the need for screened women to volunteer to serve as group facilitators with minimal compensation, the need for clinic staff to be trained to train and supervise the facilitators, and for

screened women to be motivated to attend all group sessions, and engage in ongoing advocacy. These factors are well-understood from our Exploration work, and will inform the Preparation of the clinics, to ensure that strategies are developed to address them.

C. INNOVATION There are several innovative aspects of the proposed research.
* We are unaware of any other study that has *used a network-driven approach and empowerment of CC screened peers as change agents to increase CC screening*, in any setting, including sub-Saharan Africa--despite the empirical evidence that peer support related factors facilitate CC screening uptake.

* We will examine the potential for a *multiplier effect of the advocacy training*. In the pilot, GC-CCP increased CC advocacy among intervention recipients, as well as the alters who were targeted with advocacy. Diffusion of advocacy may heighten uptake of CC screening throughout networks and larger community via increased CC knowledge, peer support, and stigma reduction. Unlike the pilot, the proposed study will assess the prevalence, quality and effects of advocacy conducted by 1st degree alters of the index participants, and the social network members of these alters (2nd degree alters), as well as explore how these intervention effects may attenuate the further the advocate is from direct exposure to the GC-CCP advocacy training.

* Most hybrid designs rely on the study team to train and supervise intervention personnel, despite the need to study implementation processes in a real-world context. We will start with the study team training facilitators to implement GC-CCP, but then *transfer training and supervision duties to clinic staff who will be trained and mentored* to take on this role for the majority of the study. This will enable us to identify barriers and facilitators to implementation and sustainability of GC-CCP across diverse (urban/rural, public/private) clinic settings.

If shown to be an effective implementation strategy for promoting health screening and other health behaviors, this peer advocacy model could be applied to other disease contexts in both LMIC and high resource settings.

D. APPROACH
The **study team** combines Ugandan experts in women's reproductive health, cancer, and health promotion interventions, with US-based researchers at RAND who developed and have evaluated the Game Changers intervention model, and have expertise in IS and social networks analysis. At Makerere University, **Wanyenze** and **Beyeza-Kashesya** are national leaders in women's reproductive health and have led CC research;[41,42] **Matovu** evaluates network-based interventions to promote health behaviors.[28,43-45] **Namisango** (from African Palliative Care Association) is an expert in palliative and cancer care, training and research in Uganda. **Nakami** leads Rays of Hope Hospice Jinja, which provides cancer care services in Uganda, and provide VIA and thermal therapy training to providers. **Wagner** has led HIV, reproductive and mental health research in Uganda over the past 25 years, including research on the Game Changers model in both the context of HIV and CC prevention.[28,46,47] **Bogart** (with Wanyenze) has conducted HIV prevention research with fisherfolk populations in Uganda for over 10 years,[48,49] and led (along with Wagner, Matovu and Green) the development and evaluation of the Game Changers intervention in the HIV context.[28] **Ober**, an IS expert, has conducted several studies of health promotion interventions using IS frameworks.[50-53] **Green** is an expert in applied network analysis and evaluation of network-based interventions.[54,55] The team includes two CC-related policy makers in Uganda: **Nakisige** oversees cancer care at Uganda Cancer Institute and is leading national efforts to increase access to CC screening and treatment; and **Mutungi** is head of Non-Communicable Diseases at the MoH. The various investigators that make up this team have collaborated on 8 prior NIH-funded grants (see biosketches for publications from this research), including the R21 pilot evaluation of GC-CCP, and R34 and R01 evaluations of GC-HIV. Wagner, Bogart and Wanyenze are the MPIs of this project: Wagner will lead the implementation and effectiveness evaluation of GC-CCP; Bogart will lead the mixed methods approach to the IS evaluation; and Wanyenze will coordinate collaboration with the various in-country partners and stakeholders, and oversee the Preparation activities and on-the-ground carrying out of the study protocol.

D.1 Preliminary Studies
We have conducted extensive research on health advocacy among social networks in Uganda, and how to mobilize people who have accessed health care to advocate for health care seeking and disease prevention within their social networks, including the development and piloting of *Game Changers*—a social network driven intervention to prepare and train individuals to engage in advocacy for health behavior—in the context of both HIV and CC prevention. This research serves as the foundation for the proposed study and is described below. Furthermore, our team has extensive experience with conducting IS research. Ober is our primary IS expert, and Bogart, Wanyenze and Wagner also have expertise in mixed methods IS evaluations and use of IS frameworks, including RE-AIM and EPIS (see biosketches).

* ***Pilot of Game Changers for HIV prevention (GC-HIV)***: Formative research in Uganda revealed that all HIV clients in care engage in some form of prevention advocacy with their social networks, but such advocacy typically involves general cautionary messages, rather than advocating specific protective behaviors, and level of engagement in advocacy was associated with lower internalized stigma and comfort with disclosure of HIV status.[56-59] With an R34 grant, we developed and piloted GC-HIV, a 6-session, peer-led group intervention in which HIV clients were trained to engage in HIV prevention advocacy with social network members (alters).[28] The RCT revealed alters of the intervention group reported less condomless sex in the past month (7.7% decrease from baseline) compared to an increase (10.5%) in the control group (p=.08; effect size = .76); and more HIV-negative alters in the intervention arm got HIV-tested (33% vs 20%; effect size=.30). Index participants (as did alters) in the intervention arm reported more prevention advocacy, less internalized HIV stigma, and greater HIV disclosure (p's < .05). We have a current R01 grant to more rigorously test GC-HIV.[47]
* ***Pilot of Game Changers for CC prevention (GC-CCP)***: With an R21, we did formative research to adapt the GC model for CC.[46,60] Focus groups with women who had and had not screened for CC revealed that like the HIV context, stigma, knowledge, and disclosure were key factors for engagement in advocacy, and thus retained in the intervention model. Internalized stigma did not appear to be an impediment to CC screening per se, but rather stigma and fear acted more as barriers to CC screening disclosure (especially if screened positive), and procedures related to VIA and treatment (e.g., misconceptions that such procedures would harm the vagina and make a woman sterile). A 7-session version of GC-CCP was developed and tested in an RCT with 40 screened women (20 intervention, 20 control) as index participants, from whom 103 alters (58 intervention, 45 control) who had never screened for CC were recruited; all participants were followed for 6 months (with 96% retention). The intervention was feasible and acceptable: peer facilitators were trained to implement the sessions, and 19/20 intervention index participants attended every session.
Intervention effects on CC screening, CC prevention advocacy, and related outcomes. In multivariate models controlling for baseline measures of the dependent variable and sociodemographics, alters in the intervention group were much more likely to have been screened for CC at month 6 [64% vs. 16%; adj. OR (95% CI) = 12.13 (4.07, 36.16)], and a higher percentage of alters named in the network assessments of the index survey (360 alters across all index participants; none of whom were perceived to have ever been screened at baseline) were perceived by the index to have been screened at month 6 [57% vs. 14%; adj. beta (SE) = 0.40 (.07); p<.001], compared to those in the control. Index participants who perceived an enrolled alter to have screened for CC at month 6, were correct 88% of time. Alters in the intervention arm also reported greater engagement in CC prevention advocacy, CC knowledge, and CC risk management, compared to control alters (all p values < .001). Intervention index participants reported greater CC prevention advocacy at month 6, as well as greater sharing of CC screening experience, CC knowledge, CC prevention advocacy self-efficacy, and CC risk management (most p values < .001), compared to index participants in the control group.

Two focus groups were held with women who completed GC-CCP. The most striking themes: (1) the training and peer support empowered women to share their personal experiences with CC screening/treatment and to engage in advocacy ("*I used to feel embarrassed to talk about my screening. But when I saw my fellow trainees come out openly, I got the boldness to also share. It gave me the freedom and courage… to share and help others learn from my experience. I don't feel any shame now."*); and (2) a strong sense of pride and passion about their new role as advocates and educators about CC and the need for screening. Many talked about wanting to engage in advocacy not only in their network, but more widely in the community, and that more and more women need to be trained, so that the dissemination of knowledge and advocacy diffusion can be as wide as possible ("*Because of the knowledge we acquired, we have a voice and people listen to us in the community. We are respected. We are 'big'. We are consulted by fellow women in case they want to learn anything about cervical cancer. So am very thankful to the facilitators. Please, train more women*").

These findings show strong effects of GC-CCP on alter CC screening uptake, providing strong justification for further evaluation of the GC-CCP as an implementation strategy to increase CC screening. The intervention effects on increased CC prevention advocacy and most components of the intervention model validates the conceptual framework of GC-CCP. Further, CC prevention advocacy also increased among alter participants in the intervention group, lending support to the advocacy diffusion potential of GC-CCP.

D.2. Study Overview. This hybrid type 1 RCT will evaluate GC-CCP for increasing CC screening, and identify barriers and facilitators to sustained implementation across diverse settings. GC-CCP is a clinical intervention that meets ERIC criteria[61] for a patient-level implementation strategy for increasing uptake of evidence-based VIA screening (see D.2.2). The EPIS framework[39,40] guides the study design. <u>Exploration:</u> With VIA screening being the EBP in the context of CC prevention, we conducted this exploratory work in our R21-funded

formative research and pilot of GC-CCP. In the proposed study, we continue at the phase of Preparation: In Year 1, we will identify modifiable clinic and provider level barriers to easy access to CC screening at 4 study clinics [2 public, 2 private-not-for-profit (PNFP), one each in an urban and rural location]. To prepare the clinics for implementation, we will conduct feasible remediation of barriers and enhance usual care via provider training on the importance of recommending CC screening to clients. Implementation: At each clinic, 40 women screened for CC will be enrolled in the RCT as index participants (n=160 index) and randomized to the intervention (in two groups of 10) or wait-list control. Each index will recruit up to three (1st degree) alters (n~440 alters) at baseline who have not screened for CC; these index and alter participants will be followed up at months 6 and 12. At month 6, these alters will each recruit up to two (2nd degree) alters (n~800 alters) for a single phone interview. Sustainment: After the enrolled index participants receive the intervention, each clinic will continue to implement GC-CCP with clients not enrolled in the trial on a quarterly basis for an additional 2.5 years, with training and supervision tasks transferred to clinic staff. Using the RE-AIM framework,[62] we will evaluate engagement in GC-CCP and CC advocacy (reach), alter CC screening (effectiveness), adoption into clinic operations, implementation outcomes (acceptability, feasibility, fidelity) and maintenance.

Study sites. To evaluate the effectiveness of GC-CCP as an implementation strategy for increasing CC screening across varied settings, the trial will be conducted in 4 clinics—Nsambya Hospital (PNFP) and Kawempe National Referral Hospital (public) in urban Kampala, and St. Charles Lwanga Hospital (PNFP) and Kayunga Regional Referral Hospital (public) near the rural town of Jinja. The sites vary by urbanicity and funding source, which may influence challenges related to GC-CCP implementation and sustainment (e.g., client awareness and attitudes towards CC screening; availability of clinic resources). Also, public and PNFP clinics are each utilized by ~40% of the Ugandan population,[77] so representation from both sectors bolsters the study's external validity. All sites are committed to collaborating with the project team (see letters of support) and have providers willing to increase availability of CC screening, and be trained and mentored to take up the GC-CCP training and supervisory role in the Sustainment Phase. All sites offer VIA screening (range: 6-30 women per day), by 6-10 trained staff; all women who request screening receive the service. Two sites (Kayunga, Kawempe) conduct thermal therapy and one site (Nsambya) conducts cryotherapy (because it doesn't have a thermal coagulator); St. Charles Lwanga does not provide treatment because it lacks a thermal coagulator. Both Nsambya and St. Charles are committed to providing thermal therapy once the project provides the needed thermal coagulator and training in the Preparation phase. Three sites (Nsambya, Kayunga, Kawempe) conduct biopsies and pap smears (for menopausal women); St. Charles does not offer these services. The staff at all sites have been trained in the use of continuous quality improvement principles and practices, and thus will have familiarity with the type of site Preparation activities described below.

D.2.1 PREPARATION of sites for implementing GC-CCP includes identification and remediation of clinic and provider level barriers to easy access to and delivery of CC screening. Qualitative methods will be used to assess knowledge of and barriers to CC screening among clinic leadership, providers and clients to ensure that all participating clinics provide good access to CC screening and thermal therapy, and to address key modifiable barriers prior to the Implementation Phase using a Plan-Do-Check-Act process[63-66] to make these improvements. This Preparation phase is key as GC-CCP may result in high demand for CC screening, but clinic-level barriers could impede the "supply" of CC screening. We will conduct semi-structured interviews and focus groups to glean data on capacity, knowledge and skill gaps concerning CC screening, drawing from the EPIS framework[39,40] (see Figure 2) to assess: Bridging factors (collaboration between clinic leadership and researchers; communication between the MoH and clinic leadership); Inner Context factors (provider knowledge and skills and clinic capacity); Innovation factors (knowledge of the effectiveness of CC screening and perceived appropriateness, acceptability and feasibility); and Outer context factors (client factors; national and local CC screening directives and trends). We also will assess overall support for and barriers to GC-CCP as part of the process to ensure successful implementation.

Semi-structured interviews. Across the 4 sites, we will conduct semi-structured interviews with leadership (n ~12 total), and relevant providers (n ~12 total), as well as MoH and local health official policymakers (n ~ 6). Interviewers will attend clinic staff meetings to introduce the study and offer participation. ***Focus Groups.*** We will conduct 8 focus groups with female clients aged 25-49 (2 per clinic in the 4 clinics; 8-10 per group). Focus groups were effective in the pilot, allowing clients to exchange ideas about barriers to CC screening and GC-CCP, some of which may be unique to the participating clinics (compared to those in our pilot). Participants will be recruited by clinic staff. All participants will be given 40,000 Ush (~$10). (Sample sizes are based on recommendations for attaining saturation in homogeneous subpopulations.)[67]

Semi-structured Interview Guide. Matovu and Beyeza will conduct the interviews. An interview guide based on EPIS domains[39,40] and recommendations for implementation research outcomes (appropriateness, acceptability, feasibility)[68] will be used (see Table 1 for examples of questions to be asked). Interviews will start with a general "grand-tour" questions, followed by more focused questions to elicit in-depth information about provider and clinic barriers to CC screening and GC-CCP implementation; proposed solutions to barriers; and feedback on the program's fit with the clinic.[69] (We will ask similar questions during the Sustainment Phase, and ask the extent to which different elements have changed since the start of the GC-CCP intervention study.)

Table 1: Sample Provider Semi-Structured Interview Guide

EPIS Domain	Construct	Sample Questions/Measures
**Bridging Factors**	* Leadership support<br>* Relationships	** To what extent does your clinic leadership support CC screening? How does that affect implementation?<br>* To what extent are [have] you been you willing to work on a project with researchers from outside of the clinic? How might [does] research affect clinic workflow?<br>* What partnerships (e.g., with NGOs) typically help or hinder implementation of practices at this clinical generally? CC screening specifically?<br>* What other relationships in/out of this clinic [could] help or hinder CC screening [GC-CCP] implementation?*
**Innovation**	* Effectiveness<br>* Complexity<br>* Relevance/Fit<br>* Acceptability	** How effective do you think CC-screening is [or will be]? GC-CCP?<br>* How easy or hard is the process of conducting CC screening? GC-CCP?<br>* How does CC screening fit with your work duties? GC-CCP? How might they detract?<br>* What are your perceptions of the feasibility of CC screening? GC-CCP?*
**Inner Context**	* Provider Knowledge<br>* Provider Self-efficacy<br>* Provider Motivation<br>* Appropriateness<br>* Feasibility	** How confident are you in conducting CC screening? GC-CCP? Why/why not?<br>* How willing are you to conduct CC screening [GC-CCP] with all female patients? Why/why not?<br>* How prepared (or not) are you to conduct CC screening [GC-CCP] for all female patients? Why/why not?<br>* How much have you had to turn women away who wanted to be screened? Why?<br>* What challenges might/did affect implementation of GC-CCP in your clinic?<br>* How do you think that GC-CCP could/did affect clinic flow? …increase your workload?<br>* How much is staffing/resources an issue? How could these barriers be addressed?<br>* What kind of [added] training do you think GC-CCP peer facilitators need?*
**Outer Context**	* Local /national regulations and requirements<br>* Client characteristics	** How do Ministry of Health policies affect implementation of CC screening here?<br>* How do funder policies and requirements affect CC screening?<br>* What other local or national policies affect CC screening? How so?<br>* How open are your patients to CC screening? GC-CCP participation?<br>* What patient factors impede CC screening? Participation in GC-CCP?*
**Other**	* Other barriers	** What other factors affect CC screening here? GC-CCP implementation?<br>* What main things that need to change for all clients to be CC screened? GC-CCP?<br>* What can be done to make sure CC screening [GC-CCP] continues at this clinic?*

Client Focus Group Guide. The guide will focus on the acceptability of CC screening and GC-CCP. Grand tour questions will be followed with probes and closed-ended questions: E.g., "Tell me what you have heard about the risk of cervical cancer" followed up by, "Have you heard that screening for CC is available for free to all women who come to this clinic?" "How willing are you to get screened for CC? Why/Why not?"

Qualitative Analysis. Interviews and focus groups will be audiotaped, transcribed, and translated. Using standard analysis methods,[70,71] two team members will read all transcripts to develop a list of themes for the implementation constructs, and a codebook listing each theme accompanied by a detailed description, inclusion/exclusion criteria, and examples. Using *Dedoose*, two coders will mark text corresponding to each theme. Coders will independently code a randomly selected 20% of transcripts to assess coder consistency (i.e., Kappas ≥.70). We will examine distribution of themes, overall and by participant type.[72]

Using plan-do-study-check cycles to address CC screening implementation barriers. With interview and focus group data, we will compile a list of barriers and gaps, then consistent with the continuous quality improvement, with leadership at each clinic we will use Plan-Do-Check-Act (PDCA) cycles[63-66] to address one main selected gap (e.g., low provision of client CC education). PDCA cycles offer a structured plan to engaging staff in making iterative, feedback-based changes in service delivery, which help to ensure that changes fit within an organization and can lower resistance from providers who will be affected by changes in delivery.[73]

"Planning" involves a designated implementation leader from each clinic working with the study team to identify 2-3 achievable improvements to CC screening; "Doing" implements a strategy for achieving the change (e.g., conducting a training for all staff, inviting a CC expert to speak about the importance of CC screening, writing an SOP for CC screening); "Checking" assesses changes in target measures, such as provider knowledge,

self-efficacy and practices regarding CC screening; and "Acting" involves making additional modifications to address ongoing gaps and barriers. PDCA cycles will end after Year 1 and prior to implementation of GC-CCP.

Our primary focus will be to equip the clinics with skills to address clinic, provider and client level barriers that hamper effective delivery of CC screening services. To ensure a comparable environment of access to CC services across all sites by the completion of the Preparation phase, the project will provide thermocoagulators (and any necessary provider training on thermal therapy) for the two sites that currently do not have them, so that all sites can provide thermal therapy, and other equipment or supplies needed for optimal client access to CC screening and thermal therapy. Once the Implementation phase begins, the sites will be responsible for ensuring an adequate, sustainable stock of supplies, so that the study can assess the ability of the sites (and external stakeholders, such as MoH) to manage this critical factor over time. In addition, providers at each site will participate in a one-day onsite training that will involve provision of information on CC including etiology, epidemiology, prevention and treatment, with a focus on the importance of educating clients about CC and recommending CC screening to all eligible clients, as well as communication strategies (e.g., standardized scripts) for how to convey messages about risk factors and importance of early screening to clients with empathy, not judgment or stigma. A standardized training manual will be developed to guide this process.

We will also work with the health facility leadership and all staff involved with CC service delivery at each site to form teams of 3-4 persons drawn from across departments, and instruct them in how to conduct a situational assessment—brainstorming on the "process-related" challenges affecting CC screening access and uptake at their site, coming up with a general list of problems and selecting a main problem to address, identifying the root cause of that problem, and generating strategies to address the problem or gap within 6-9 months. For example, a priority area might be the need to track clients who are screened, including those who screen positive and require follow-up, and a solution could be to institute a CC screening registry that facilitates tracking. The selected gaps may differ by site depending on what the health facility identifies as the priority problem. Site teams will implement the countermeasures over a period of 6-9 months and evaluate the changes made in access to and uptake of CC screening services at the end of this period. At the end of the Preparation phase, we will have identified mechanisms through which improvements can be achieved, and we will have created quality improvement teams at each site that we will work with during the Implementation phase. We have previous used a similar approach in addressing health facility challenges affecting the delivery of HIV/AIDS, malaria and TB programs in Uganda with impressive results.[74]

D.2.2 <u>IMPLEMENTATION</u> of GC-CCP: RCT evaluation of effectiveness in increasing CC screening

In the first 6 months of the 3-year GC-CCP implementation and data collection, the hybrid type 1 effectiveness-implementation RCT begins, and enrolled index participants receive GC-CCP. A hybrid type 1 trial[75] tests "a clinical intervention while gathering data on its delivery during the effectiveness trial, and its potential for implementation in a real-world situation." GC-CCP is a clinical intervention that also fits 2 categories of patient-level implementation strategies included in the ERIC compilation:[61] identify and prepare champions or early adopters, and intervene with and/or involve patients/consumers to enhance uptake. GC-CCP fits within these categories, as it empowers women who have screened for CC to engage in CC screening advocacy with women in their social network. Below is a further delineation of the GC-CCP implementation strategy.

**Name**	**Peer-facilitated advocacy training**	**Peer advocacy for CC prevention**
**Definition**	Peer-facilitated group training of CC screened women to engage in CC prevention advocacy	Peer advocacy for CC screening and prevention with social network members
**Specification**		
Actor(s)	a. CC screened women trained to facilitate the advocacy training<br>b. Clinic staff assigned to train and supervise peer facilitators	a. CC screened women who receive the advocacy training
Action(s)	a. Facilitation of intervention sessions<br>b. Training peer facilitators at start of program<br>c. Ongoing supervision of peer facilitators	a. Engagement in advocacy for CC prevention and screening with alters<br>b. Share CC screening experience
Action target	a. CC prevention advocacy conducted by women receiving the training<br>b. Effective facilitation of training sessions, with fidelity to the manual	a. Alters who are unscreened for CC<br>b. Uptake of CC screening among alters
Temporality	a. Peer facilitators are identified and trained to implement the advocacy group training with CC screened women<br>b. CC screened women are recruited to participate in the group advocacy training<br>c. Trained CC screened women engage in advocacy with alters over time<br>d. Alter recipients of advocacy engage in advocacy with other alters	
Dose	a. 3-day training of peer facilitators at start of program; training is renewed when there is turnover and new facilitators need to be trained<br>b. GC-CCP advocacy training occurs across 7 weekly 2-hr sessions<br>c. GC-CCP training program implemented quarterly at the clinic	a. Advocacy with a particular alter can occur once, or over several discussions<br>b. Ongoing nature of periodic screening warrants ongoing peer advocacy

The hybrid trial will collect effectiveness and implementation data, but the RCT will focus mostly on evaluating the intervention's **effectiveness** in increasing alter CC screening. However, other implementation outcomes such as **reach** (client enrollment in the GC-CCP training, and index and alter engagement in CC screening advocacy) will draw data from the research cohort enrolled in the RCT. The implementation process is initiated in this phase, but the related outcomes will be more fully evaluated in the study's Sustainability phase.

Game Changers for CC Prevention (GC-CCP). GC-CCP draws on theories of network diffusion,[19] cognitive consistency,[76] and social influence,[20] which posit that behavior change can be initiated by a few and diffused to others through modeling, advocacy, and shifts in social norms. GC-CCP uses 7 sessions to empower CC screened women to act as change agents for CC screening in their social networks. ***Session 1*** addresses fears and concerns related to CC risk and use of self-compassion and peer support to overcome these fears. ***Session 2*** focuses on decision making for sharing one's personal CC screening experience, and how to initiate disclosure and conversations about CC. ***Session 3*** builds self-efficacy for CC risk management so that one's own behavior (e.g., recognizing signs of CC risk and seeking CC-related health services as needed; periodic VIA screens) coincides with the behavior they encourage in others, as well as learning CC facts and myths to facilitate accurate CC screening advocacy. ***Session 4*** demonstrates how one's social network can serve as a tool for dissemination of CC prevention advocacy and knowledge. ***Sessions 5 and 6*** build CC advocacy skills: communication skills (e.g., reflective listening, paraphrasing, open ended questions), and how to start and sustain conversations about CC. Participants learn to identify alters in their network who may be prime targets for advocacy and transfer of information and norms throughout the network (e.g., well connected to other alters; are "bridges" between two distinct groups of alters). ***Session 7*** inspires commitment to ongoing CC advocacy through peer solidarity and support. The 2-hour, weekly sessions will be conducted in Luganda using a structured manual, and group format to facilitate: sharing of experiences to build solidarity, support, and motivation among participants; group problem solving and role playing to build skills and self-efficacy; and homework between sessions to practice new skills and generate experiences to be processed in the sessions.

Facilitator training, supervision, and fidelity monitoring. Two bilingual (Luganda, English) adult female clients of the clinic will be recruited by the clinic staff and trained as intervention peer facilitators. Criteria for facilitators include having in-depth knowledge of the local community and the experience of CC screening; they may be counselors or community health workers (trained lay persons) whose role often includes community outreach and education, and facilitating client engagement and use of health services. The training, conducted by Wagner, Bogart, Green, Mutaasa and Namisango, will include reviewing objectives for each session, step-by-step scripts, role playing and mock implementation of core exercises. Training will cover group facilitation, building rapport, reflective listening, and dealing with group conflict. The supervisors of the initial two groups at each site (i.e., the groups administered to the research index participants), Namisango and Mutaasa, will each attend each session of the initial group at two sites, and complete fidelity rating forms; each facilitator will also complete fidelity forms after each session. These forms will include ratings of fidelity to the manual, participant engagement, and quality of facilitation, and will be used to facilitate weekly supervision between sessions.

D2.2.1 Multi-site RCT of CC-CCP. At all 4 sites, 40 women screened for CC in the past year will enroll as index participants (n=160), with 20 randomly assigned to the intervention, and 20 to a wait-list usual care control. Individual randomization entails contamination risks, but statistical models suggest ≥30% of the control arm must receive the equivalent of a full-strength intervention for meaningful contamination.[78] We decided against an attention control, because attention paid to intervention (index) participants would not impact alter behavior (primary outcome), and its policy implications would be less clear. The intervention arm will be divided into 2 groups of 10 (balanced on ages 25-35 and 36+) for receipt of the intervention. The control arm receives the intervention after the 12-month follow-ups. Each index participant will recruit up to 3 alters (total= ~440 alters; referred to as 1st degree alters) with no history of CC screening. All index and 1st degree alter participants will be followed up at months 6 and 12. At month 6, 1st degree alters will recruit up to 2 of their own alters (2nd degree alters; total= ~800) who they report targeting with advocacy and thereafter getting screened for CC; 2nd degree alters will be administered a single, brief phone interview.

Eligibility criteria: *Index participants:* female, age 25+ (the lower limit for recommended CC screening), screened for CC in past year (clinic records), and no advanced stage disease (12-month follow-up is feasible). *1st degree alters:* female, age 25+, and report no CC screening history. *2nd degree alters:* female and age 25+.

Recruitment of index and 1st degree alters will span 8 weeks (5 index and 14 alters per week) at each site. Clinic staff will contact eligible women who have been screened for CC in the past year to inform them of the study; if interested, the site coordinator will perform consent processes and formal eligibility screening. Eligible women who enroll will complete the baseline survey, followed by randomization as an index participant.

1st degree alters: The site coordinator will use the social network assessment within the baseline survey of each index to randomly select (using a random number table) 5 alters who know the participant's CC screening experience (or as many as there are if < 5) and ask the index if she is comfortable recruiting 3 of these alters. The index will be asked to call each selected alter at the end of their baseline interview to describe the study in the presence of the coordinator (or later in private, if she prefers), who will schedule a study visit for the alter. If an alter refuses or cannot be reached, a replacement will be randomly chosen from the original list of 5 alters.
2nd degree alters: The site coordinator will use the social network assessment in the month 6 survey of each 1st degree alter to randomly select 4 alters (or as many as there are if < 4) who the alter reports targeting with CC screening advocacy and thereafter was screened for CC. A similar process as described above for 1st degree alters, will be used to recruit and enroll 2nd degree alters. The coordinator will obtain verbal consent from the 2nd degree alter over the phone and the complete a brief 15-minute phone survey.

Assessment schedule: Surveys will be administered at baseline, month 6 and month 12 for index and 1st degree alters, and 2nd degree alters will be surveyed only once (at month 6 assessment of 1st degree alters). Participants will receive 30,000 Ush (~$8) per assessment, except 2nd degree alters who will receive 10,000 Ush (via mobile money). To limit attrition, we will collect tracking information (phone numbers and mapped addresses), including contacts for family/friends with whom they have frequent contact. Clinic staff will help us track clients we have difficulty reaching. In the pilot, >96% of index and alters were retained at month 6.

Measures: Survey assessments for index and 1st degree alter participants will be interviewer-administered in Luganda using Network Canvas software, last ~ 75 minutes, and include a social network assessment. CC screening and treatment utilization will be verified with medical chart data.

**Survey Constructs**	**Instrument**
Primary Outcome	
CC screening (VIA) and results	Survey; medical chart
Secondary Outcomes	
CC-related treatment (e.g., cryotherapy, thermal therapy; radiation); biopsy	Survey; medical chart
CC prevention and treatment advocacy (general)	6 items; dev. In- house; alpha=.95
CC screening advocacy with specific alter (discussed/encouraged/gave info/direct support)	4 items; dev. In-house; alpha=.87
CC screening advocacy received from index as perceived by alter (discussed/encouraged/gave info/direct support) (alter survey only)	4 items; dev. In-house; alpha=.86
Potential Mediators	
CC knowledge (etiology, prevention, treatment)	16 items; dev. in-house; alpha=.75
CC internalized stigma	5 items; adapted from Kalichman[79]
CC enacted stigma	6 items; adapted from Marlow & Wardle[80] and Cho et al.[81]
Extent of sharing of CC screening experience and result	3 items; dev. In-house; alpha=.74
CC prevention advocacy self-efficacy	3 items; dev. In-house; alpha=.85
CC risk management self-efficacy (i.e., can identify signs of risk and seek help)	3 items; dev. In-house; alpha=.64
Covariates/Potential Moderators	
Background characteristics (age, education, income, relationship status, HIV status)	Standard items
General social support	4 items from MOS (Moser et al)[82]
Partner support for CC screening and treatment	4 items; dev. In-house; alpha=.83

Social network assessment: The participant will list 10 female alters (which is adequate for structural and compositional variability)[83] with whom they interact most. For each listed alter, we will gather information to assess *network composition* (e.g., age, relation to respondent, history of CC screening and treatment; knowledge of respondent's CC screening experience). In the index survey only, we will ask how if each alter knows each other alter to assess *network structure* (e.g., density); this section is time consuming, so given the large number of alter participants, alters will not complete this survey component. Research questions related to this section [e.g., do denser networks promote more CC screening within the network) will be addressed with index participant data. *To assess CC advocacy and perceived effects*, from the perspective of both the index and alter, we will ask the participants if they have engaged in CC screening advocacy (separate items for discussed, encouraged, gave info, gave direct support, and frequency) with the specific alter in the past 6 months, and any perceived resulting action (e.g., alter was screened). At follow-up, we will determine whether listed alters are the same or unique from those listed at baseline, to facilitate longitudinal analyses of alter data.

Phone assessment of 2nd degree alters will assess background characteristics, receipt of CC screening and treatment services, CC knowledge, receipt of CC screening advocacy from referring 1st degree alter, engagement in CC prevention advocacy (general), number of alters engaged in CC screening advocacy with, and the number of these alters perceived to have been screened for CC following this advocacy.

Statistical Analysis

Power: Assuming 220 1st degree alters per arm enroll, 10% attrition (month 6 retention in pilot was 96%; we have retained 90-94% over 12 months in past studies in Uganda),[84,85] ICC=.01-.05 range to control for clustering within the alters of each index participant, and a 15% CC screening rate in control alters (based on pilot data): the effective sample size will be 200 (ICC=.05) or 216 (ICC=.01) per arm. If ICC=.01, we will be able to detect a small effect size (11 percentage point difference) in alter CC screening at month 12 (80% power; alpha = .05). If ICC=.05, the detectable effect size is 12 percentage point difference (or odds ratio of 2.0).

Aim 1: *Assess effects of GC-CCP on alter CC screening.* We will use an intent-to-treat approach. In addition to comparing the arms at baseline and months 6 and 12, we will apply logistic generalized mixed models to our repeated-measures data to examine intervention effects, using a time by arm interaction to assess differences between the arms over time. We will account for correlation among participants in the same intervention group, and among alters referred by the same index, by adjusting standard errors for statistical inference tests with a sandwich estimator.[86-93] We will use imputation for item nonresponse and attrition weights to account for non-random dropouts using logistic regression. We will control for and examine interaction effects with index (e.g., age), alter (e.g., knowledge of index's CC screening), and intervention (sessions completed) variables.
Aim 2: The analysis for this aim is described in the Preparation and Sustainment phases.
Aim 3: *Examine mediators and moderators of the intervention effects on alter CC screening, and index and alter engagement in CC prevention advocacy.* Potential mediators consist of intervention mechanisms (sharing of CC screening experience, CC knowledge, CC risk management, CC prevention advocacy self-efficacy), while potential moderators include age, history of CC treatment, and network characteristics (e.g., % of alters who are trustworthy). We will explore interactions between a moderator and study arm to examine if the variable moderates the intervention effects. If categorical moderators are significant, we will conduct post-hoc simple main effects t-tests with Bonferroni corrections. For continuous moderators,[94] we will graph significant interactions at low and high levels of each score (±1 SD) and conduct post-hoc simple main effects t-tests with Bonferroni correction by comparing differences between discrete points on the regression lines. To test for mediation, we will run regression models where: (a) the outcome is CC screening measured at month 12 and predictors are intervention indicator, a potential mediator measured at month 6, and covariates, if any; and (b) the outcome is the same potential mediator measured at month 6 and predictors are the intervention indicator and covariates, if any. We will apply a bootstrapping approach to the results from those two models to test whether mediators account for effects,[96,97] using SAS Proc Causalmed.

Aim 4. *Evaluate the cost and cost-effectiveness of implementing GC-CCP to increase CC screening and early-stage treatment*. We will compare *GC-CCP* vs. enhanced usual care on the marginal cost of increasing alter CC screening.[98] Following standard convention,[98] we will define the incremental cost-effectiveness ratio (ICER) as the difference in per-capita cost of the intervention versus control group divided by the difference in their average effectiveness: $ICER = \frac{\mu_{C2}-\mu_{C1}}{\delta_{e2}-\delta_{e1}}$ (μ_{c2} = mean per-capita cost of *GC-CCP*, μ_{c1} = mean per-capita cost of usual care, δ_{e2} = % of alters who get screened in GC-CCP, δ_{e1} = % of alters who get screened in enhanced usual care. We will estimate confidence intervals with bootstrap methods.[99] We will examine economic costs from a societal perspective, incorporating estimates of the frequency and duration of time spent by individuals engaging in receipt of services, as well as transportation costs and forgone employment as relevant. We will use a micro-costing approach, time-driven activity based costing (TDABC), to track all costs associated with implementing *GC-CCP* and enhanced usual care as estimated from data collected by the sites and study team.[100] The cost per resource will be calculated by multiplying the quantity used by unit cost; total cost will be derived by summating individual costs.[101] Capital costs will be annualized using a discount rate of 3% with an assumed lifespan of 30 (buildings) and 10 (furniture) years.[101] Facilitators will record time spent on the sessions, identifying each activity (e.g., training, preparation, in-session) and session-related materials (e.g., consumable materials). Unit costs for labor will be characterized as capacity cost rates, as described in standard TDABC methodology. Intervention development (training) and ongoing costs will be differentiated; ongoing costs will be tracked to determine cost efficiencies over time, and we will differentiate fixed from variable intervention costs to assess the marginal cost of providing care to additional clients.

D.2.3 SUSTAINMENT: Evaluation of GC-CCP implementation and maintenance process and outcomes
After implementing GC-CCP with research enrolled index participants in the first 6 months of implementation to evaluate effectiveness, clinic staff will take over all aspects of implementation during the Sustainment phase (2.5 years). The study team will transition the training and supervision of peer facilitators to clinic providers. This phase is important to (a) fully understand implementation processes, facilitators of, and barriers to real-world implementation of GC-CCP (without research staff); (b) to study sustainability of GC-CCP (as well as CC prevention advocacy and CC screening) when GC-CCP is fully implemented by clinic staff; and (c) to prepare the clinics to maintain GC-CPP into the future, if it is found to be cost-effective in the RCT.

Identifying and training clinic staff to supervise GC-CPP. The study team will work with the clinics to identify staff to serve as supervisors for the Sustainment phase. The selected staff member will likely vary from site to site based on variability in staff composition and skill sets, but female counselors and nurses are suitable, if they have experience supervising other personnel. The peer facilitators will be identified and trained at the start of the Implementation phase (see D.2.2), but there is likely to be some turnover and need for training of new facilitators at some point during the 2.5 years of Sustainment. The clinic staff who will train and supervise the peer facilitators will be identified at the start of the RCT, so that they can participate in the initial training of facilitators as a co-trainer, receiving mentorship from the study team trainers and supervisors; she will also shadow Mutaasa and Namisango as they carry out supervision (including completion of fidelity rating forms after observing the implementation of sessions), in order to receive mentorship in this role. In the initial supervision sessions conducted by the staff member in the Sustainment phase, either Namisango or Mutaasa will attend twice a month for 3 months to provide continued mentorship. Supervision in this phase will be held after each session for the first group they oversee, followed by after every other session for successive groups.

RE-AIM framework outcomes will be assessed to evaluate GC-CCP implementation. Effectiveness of increasing CC screening (primary RCT outcome) will be based on chart data from enrolled alter participants. Reach in terms of how many women engage in GC-CCP and CC prevention advocacy will be assessed using chart data from CC screened clients, and survey data from enrolled index and alter participants, respectively. Semi-structured interviews and surveys with clinic staff (directors/managers, providers) and clients; supervisor ratings of facilitators (fidelity); and clinic budget ledgers (cost), will be used to assess EPIS constructs.[62,68]

Table 2. RE-AIM Outcomes

Outcome	Operationalization	Data Source & Study Phase
**Reach** (CC screened women)	a) Proportion and characteristics (e.g., age, HIV status) of CC screened women who enroll in GC-CCP; and attend >3 sessions<br>*Benchmark of success will be at least 15% of CC screened women annually who receive over half of sessions, based on Diffusion of Innovation theory, which posits that ~15% of a target group should be exposed for optimal population diffusion of information*[19,102]	Data (enrollment, attendance dates) collected by clinic staff and added to medical chart (Implementation and Sustainment)
**Reach** (CC screened women and their alters)	b) Proportion and characteristics of research enrolled women who receive GC-CCP (i.e., index participants) who engage in advocacy<br>c) Proportion and characteristics of research enrolled alters who received advocacy from index participant<br>d) Proportion and characteristics of research enrolled alters who engage in advocacy with other alters	Survey data collected during RCT; intervention vs control comparison (Implementation)
**Effectiveness** (alters)	Proportion of previously unscreened alters who get screened for CC,	Med chart (intervention vs control group in RCT; Implementation)
**Adoption** (Clinic staff)	Clinic staff integrate GC-CCP (i.e., extent to which staff identify, train, and supervise peer facilitators, recruit clients, conduct sessions):<br>• Proportion of staff who report recruiting clients for GC-CCP<br>• Extent to which staff supervisor carries out weekly supervision of peer facilitators conducting GC-CCP sessions	Twice yearly survey of clinic staff implementation activities (Sustainment)<br>Clinic staff supervisor fidelity forms (Sustainment)
**Implementation** (Fidelity)	Fidelity: Intervention facilitated as prescribed in manual (75% of session topics facilitated with fidelity across sessions)	Fidelity ratings (based on independent observations by 2 team members; Implementation & Sustainment)
**Implementation** (Perceptions of Implementation and Sustainment; EPIS domains)	a) Perceived acceptability (satisfaction with GC-CCP) (EPIS Innovation)<br>b) Appropriateness (perceived fit/compatibility of GC-CCP with current practices) (EPIS Innovation & Inner Context factors)<br>c) Feasibility (extent to which GC-CCP can be successfully conducted by clinics staff) (EPIS Innovation & Inner Context factors)<br>d) Other barriers to and facilitators of implementation	Semi-structured interviews with stakeholders (policy, clinic, and client levels); (Implementation & Sustainment)<br>Brief surveys with health care providers, using adapted measures[103] (Implementation & Sustainment)
**Implementation** (Adaptation)	Documentation of adaptations and recommendations needed to promote and sustain implementation	Semi-structured interviews (clinic staff) (Implementation & Sustainment)
**Implementation** (Cost)	Cost of implementation strategy	Clinic cost ledger (e.g., staff salaries, materials, space rental) (Sustainment)
**Maintenance** (assessed in "Sustainment" Phase only, last 2.5 years of data collection)	Implementation maintained over 2.5 years (i.e., GC-CCP conducted with at least 15% of women screened for CC; may be revised based on Implementation Phase evaluation)	Clinic records of GC-CCP implementation and client engagement (Sustainment)
	Supervision support for peer facilitators maintained over 2.5 years	Completed fidelity forms by clinic supervisor (Sustainment)
	Women receiving the intervention sustain advocacy 12-months post GC-CCP participation	Index client survey during RCT (Implementation & Sustainment)

Medical chart abstraction. We will use medical chart abstraction to assess Effectiveness (Implementation) and Reach (Sustainment). At the beginning of the RCT (Implementation Phase), we will work with the clinic to add a field to the client visit form to record referral source for CC screening (e.g., healthcare provider, another client, with names recorded); and if relevant, RCT enrollment (and date) and participation in GC-CCP (dates of sessions attended). Data on referral source will be checked for names of GC-CCP participants. From these data, we will determine Effectiveness (along with survey and chart data from enrolled alter participants) and Reach as operationalized in Table 2. Note that we have previously worked with Ugandan public and private healthcare facilities to add implementation variables to the medical chart, by creating brief hard copy forms or registries for clinic staff to complete, that can be linked with patient medical records.[48,104]
Survey data (index and alter participants). Follow-up survey data from enrolled index and alter participants will be collected during the RCT; these data will inform Maintenance in terms of sustained engagement in CC prevention advocacy (during the Sustainment phase), as noted in Table 2.
Semi-structured interviews. We will assess implementation qualitatively with semi-structured interviews with clinic staff who are involved in CC screening and GC-CCP (e.g., clinic manager, GC-CCP peer facilitator, CC screener/treater). We will interview 20 staff (5 per clinic) at a private space at the clinic at the beginning of Sustainment, followed by 15 and 30 months later (ideally, all with the same staff), to complement the quantitative outcomes in helping to understand the implementation process. Staff will be assured of confidentiality and that their responses will not be shared with others at the workplace. We will use the interview guide developed for the Preparation Phase, revising questions as needed to ask about current (vs future) practices and adding probes about specific barriers that emerged in prior interviews, to assess changes in barriers over time. Such an iterative process allows for a deeper understanding of both the past and the present.[105] We also will ask what resources are needed to sustain CC screening and GC-CCP.
Client focus groups. Based on medical chart data on GC-CCP participation and CC screening referrals, a random sample of clients who participate in GC-CCP, and a random sample of CC screened alter clients who state that they were referred by a peer who attended GC-CCP, will be asked to participate in one of two focus groups per site. Selected women will be stratified by age (age 25-35 vs. 36-49). Client participants will be referred by clinic staff to the study team for consent processes. Examples of questions to be asked of GC-CCP participants include, *How easy or challenging was it for you to engage with the training sessions (follow up: time of day, getting to training location, support from partner)? How useful was the content of the training? How much were your questions about disclosure [advocacy] addressed? How much, if at all, do you think the sessions increased your advocacy to other women?* Examples of questions to be asked of alters include, *Following the advocacy you received, what additional questions about CC screening do you have? How much were your questions about CC screening addressed? How would you describe the process of getting screened for CC? How much, if at all, do you think the advocacy you received affected your decision to get screened? How much, if at all, are you motivated to advocate as well to other women? Why/why not?*

Survey data (clinic staff). To complement the qualitative data on implementation and sustainability factors, we will collect brief survey data from clinic staff once at the beginning and once at end of the 30-month Sustainment phase. These data will include adapted survey items[103] from three companion measures focused on the fit of GC-CCP with the clinic operations (e.g., "I welcome GC-CCP into the clinic," "GC-CCP is a good fit with clinic practices," and "It is possible to offer GC-CCP," from 1=completely disagree to 5=completely agree). The questions will be tailored to the Ugandan clinic contexts during the Preparation stage of the project.

To assess Adoption, clinic staff will be given a quarterly anonymous brief survey to assess if they helped to implement GC-CCP and if so, the extent to which they performed key activities (e.g., identification and recruitment of CC screened clients to participate in GC-CCP; training or supervision of facilitators). A secure anonymous survey link will be emailed to providers, or given a hard copy to complete and insert into an envelope. We will convene clinic providers and leadership quarterly to assess implementation challenges (re. CC screening and GC-CCP) that are raised in the surveys or during the meeting, and how to address them.

Qualitative analysis methods will be similar to the Preparation phase (D.2.1). A longitudinal qualitative analysis will compare themes within and across timepoints to determine change in barriers over time (e.g., using summative analytic memos about themes at each timepoint).[105] We will triangulate complementary data collected from medical charts, semi-structured interviews and brief clinic surveys on CC screening uptake using a mixed methods approach.[106] E.g., if we find high uptake of GC-CCP among CC screened women (with >3 sessions attended), we will use the qualitative data to help explain why women found the intervention to be acceptable (e.g., that they enjoyed learning new skills for advocacy). Conversely, if we find that women are not engaging in advocacy, we may determine an explanation from the qualitative interview data (e.g., high stigma).

E. HUMAN SUBJECTS PROTECTION

1. Characteristics of Potential Subjects

We plan to enroll 1400 participants in the RCT component of the study (160 index, 440 1st degree social network members (alters), 800 2nd degree alters), plus participants in the semi-structured interviews (Preparation phase: 30 providers/clinic heads/policy makers; Sustainment phase: 20 providers) and focus groups (80 clients in both the Preparation and Sustainment phases). All client participants (RCT and focus groups) will be women age 25 years and older and of all racial (though nearly all will be Black) and tribal/ethnic backgrounds; index client participants will be required to have evidence of being screened for cervical cancer (CC) in the past year.

2. Data Sources include self-report survey and social network data, chart abstracted medical data, audio recordings of semi-structured interviews and focus group discussions, and facilitator and supervisor ratings of intervention sessions.

3. Potential Risks to Client Participants

Psychological distress: Distress may result from issues raised during focus groups, survey interviews or intervention sessions.

Breach of confidentiality: Violation of participant confidentiality is possible, but we believe the safeguards that we have integrated into the study methodology, as described below, will effectively limit this risk.

Harm from CC disclosure or advocacy: There is a risk that participation in the intervention sessions could result in the participant disclosing their CC risk status or advocating CC prevention with others and that such behavior could lead to negative responses from the recipient of such information, including discrimination, hostility, social rejection, and abandonment.

Inadvertent CC risk disclosure when asking a social network member to participate in the study: Asking a member of one's social network (e.g., family member or friend) to participate in the study could lead that person to assume that the participant has CC risk, if they don't know this already, given the nature of the study.

4. Adequacy of Protection Against Risks

Informed Consent: During the consent process, potential participants will receive a detailed description of the study, including the nature of the participant's involvement in the study, the possible risks and benefits of participation, and the participant's ability to withdraw from the study at any time without consequence. The participant will have the opportunity to ask questions about the study, after which informed consent will be obtained. Each participant will be given a copy of the consent form to keep. Written consent will be obtained from client participants, and verbal consent from providers, clinic heads and policy makers.

Index client participants will be informed of the study by clinic staff and referred to the study coordinator for consent procedures if interested in participating; index participants will recruit members of their social networks for the alter client participants. Providers, clinic heads and policy makers will be approached by study coordinators to consider study participation.

Protection Against Risk

Psychological Distress. Client participants will be referred to a counselor at the clinic if warranted and/or desired by the participant. Participants also have the right to refuse to answer specific questions and can end their participation in the group or workshop at any time.

Confidentiality. To protect confidentiality, all research data will be kept in locked file cabinets and will be available only to members of the study team. Data will be identifiable only by study numbers and participant initials. CC status will not appear along with any personal identifying information. Personal information including subject's name, address, and phone number will be stored separately from all research data. All data collected from participants will be kept confidential and not shared with clinic staff, or any of their social network members who are participating in the study. Data collected from index participants will not be shared with their participating alters, and data collected from alters will not be shared with the index participants who recruited them. Participants will be informed of these conditions during the informed consent process. Also, prior to the initiation of focus groups and intervention sessions, the importance of and commitment to maintaining the confidentiality of all that is shared in the group will be emphasized. These procedures are critical to establishing a safe environment for participants to feel comfortable being open and candid. Audio recordings of the focus groups and semi-structured interviews will have no identifying information, will be

stored on a password-protected, encrypted file, and then destroyed once a transcript has been completed (within one week of administration). All study staff will receive training on confidentiality and sign Confidentiality Agreements.

Harm from CC risk disclosure or advocacy. Among the goals of the intervention sessions are to improve the participants' ability to make effective decisions regarding disclosure of their CC risk status and engaging in CC prevention discussions with members of their social network. These sessions will be designed to help participants recognize the full range of responses that they may encounter when disclosing their status or discussing CC, including the potential for recipients of such information to act with hostility, discrimination, or other negatives responses. Hence, the sessions focus on decision making regarding these behaviors, and improving judgment for when such behaviors will be beneficial versus harmful, rather than encouraging all participants to disclose or advocate prevention regardless of risk for harm. These procedures cannot prevent all negative responses, but should minimize such risks. In the pilot of the intervention, no adverse events were reported in this regard.

Inadvertent CC risk disclosure to recruited social network member: Index participants will be asked to consider recruiting up to 3 of their network members to participate in the study survey, and 1st degree alters will be asked to recruit up to 2 of their social network members. Participants will be informed that this is not a requirement and that they should only ask people who they are comfortable with approaching to participate. These participants will be asked to consider selecting these network members from a list of 5 randomly selected network members (generated by the study) whom they report know the participant's CC screening experience/result (if there are less than 5 named members who know the participant's CC screening experience/result, then only those who do know will be selected). This procedure should limit the risk of the participant trying to recruit a network member who does not know the participant's CC screening experience/result, but participants are allowed to recruit network members who are not on the list of 5 members provided to them by the study, so it is possible. However, participants will be counseled on the risks of approaching such a network member, as someone who does not know the index participant's CC screening experience could assume the index participant has CC or CC risk given the nature of the study.

5. **Potential Benefits to Study Participants:** The intervention may help clients to have better knowledge about CC risk and importance of screening, to manage any stigma related to CC risk, to make better decisions about when and to whom to share their CC screening experience, and engage in effective CC prevention advocacy. Participants will be compensated for participation, and this will vary depending on their time commitment: ~$8 (~30,000 Ugx) for completing a survey assessment, semi-structured interview or focus group discussion. 2nd degree alters will be compensated ~$3 (~10,000Ugx) as they will only engage in a brief phone survey unlike the index and 1st degree alters that will engage in a longer survey and will need to come to the clinic.

6. **Importance of the Knowledge to Be Gained:** The study will provide information about the potential impact of the peer-led advocacy training intervention for empowering women screened for CC to act as change agents for increasing CC screening uptake within their social networks.

F. DATA SAFETY AND MONITORING PLAN

I. Description of Study and Study Data

This study is a partnership among RAND Corporation (Glenn Wagner and Laura Bogart, MPIs), Makerere School of Public Health (Rhoda Wanyenze, MPI), African Palliative Care Association, Rays of Hope Hospice Jinja, and Indiana University at Bloomington. We will conduct a hybrid implementation-effectiveness randomized controlled trial (RCT) of *Game Changers for Cervical Cancer Prevention (GC-CCP)*, a 7-session peer-led group intervention that aims to empower and mobilize women who have screened for cervical cancer (CC) to be agents for CC screening and prevention in their social networks. For the RCT, we will recruit 160 screened women (index participants) across 4 clinics, randomizing 80 to the intervention and 80 to the usual care control condition. Each index participant will be asked to recruit up to 3 social network members (1st degree alters) who have never screened for CC to complete assessments (~440 total based on average of 2.75 alters recruited per index participant, 220/arm) over 12 months, to test intervention effects on alter uptake of CC screening. All participants will complete surveys at baseline, and 6- and 12-months post-baseline. At the

month 6 follow-up, first degree alters will be asked to recruit up to 2 of their social network members (2nd degree alters; ~ 800 total). Surveys of index and alter participants will include questions about CC screening, CC knowledge, CC stigma, and engagement in CC prevention advocacy. Data on alter CC screening and treatment will be extracted from medical records. All data will be collected and stored at the participating clinic sites initially, and then transferred to Makerere University School of Public Health; de-identified data will be transferred to RAND for data management and analysis.

II. Plan for Independent Data Safety and Monitoring

Data Integrity: To ensure data integrity and confidentiality, all study staff will be trained to promote standardized and objective collection and recording of participant information, and will sign Confidentiality Agreements. All data will be stored in password-protected, encrypted files that are backed up through a secure offsite connection. Audio recordings of the focus groups and semi-structured interviews will have no identifying information, will be stored on a password-protected, and encrypted file, and then destroyed once a transcript has been completed (within one week of administration).

Routine monitoring of adverse events: With regard to harm from CC risk disclosure, advocacy, or social network recruitment, we will monitor the occurrence of such events during the intervention sessions and assessments, and encourage clients to inform the facilitators of such events. Counseling will be provided to participants when these events occur. We will gauge the extent to which the level of occurrence of such events is being provoked by the sessions at a higher rate than would be expected in the absence of the intervention. This information will inform modifications to the sessions or discontinuation of the intervention. Note that no such adverse events were reported in the pilot of the intervention.

As we have did in our R21 pilot, the PI (Wagner) will monitor the data frequently (e.g., weekly), working with the Project Director, site coordinators and study statistician to obtain reports that include participant enrollment, participant retention, number of participants who drop out of or are withdrawn from the study with reasons for dropping out or withdrawing, and a list of all adverse events (AEs) that are plausibly related to the intervention or other study procedures. By monitoring data and study progress weekly, we believe the we will be able to efficiently respond to issues as they arise.

Data Safety Monitoring Board. In order to ensure and maintain the scientific integrity of this human subjects research project and to protect the safety of its research participants, we will assemble a Data Safety Monitoring Board (DSMB). The DSMB will have the responsibility of assuring that participants are not being exposed to unnecessary or unreasonable risks as a result of the pursuit of the study's scientific objectives.

Appointment. The DSMB will be composed of three individuals (2 in Uganda, 1 in the U.S.) with pertinent research expertise and knowledge about the design, monitoring, and analysis of health behavior intervention research necessary to conduct an ethical and scientifically rigorous study. At least one will have strong statistical/quantitative skills. Members will be appointed by consensus of the senior investigators. The 3-member panel will appoint one member to be Chair of the DSMB. The DSMB oversight of the study will begin prior to the initiation of participant recruitment and data collection.

Duties and Responsibilities. The DSMB will review the study protocol and any protocol changes in relation to the safety of participants and in relation to the overall scientific integrity of the project. Comments and recommendations will be directed to the MPIs. The DSMB will be charged with reviewing data integrity, participant safety, and study conduct and progress, and for providing directives regarding study continuations, modifications, and terminations. The DSMB will meet annually; however, AEs that are considered directly related to study participation will be reported immediately to the DSMB, the IRBs, and NIH. The DSMB will be provided with reports during the annual meeting that include participant enrollment, participant retention, number of patients who drop out of the study with reasons for drop out, and a listing of all AEs that are plausibly related to the intervention or other study procedures. After reviewing and discussing this information at the annual meeting, the DSMB may ask for clarification or additional information from the MPIs. At the close of each meeting, the DSMB will vote to recommend continuation, suspension, or termination of the study; the written determination and summaries of the meetings will be shared with NIH as part of the annual progress report. The DSMB will be required to exclude from meeting summaries any information that would compromise the privacy or confidentiality afforded research participants and exclude any information that could compromise the scientific integrity of the trial.

In its deliberations, the DSMB will attempt to reach a consensus before making any recommendation. The

DSMB must carefully weigh all information available and give due consideration to the ongoing balance between risk to the participant and possible scientific benefits. When this balance is not clear, or when there is persistent uncertainty or doubt, the DSMB recommendations are to reflect the principle that human safety takes precedence over scientific objectives. Recommendations made to the investigative team should represent the collective judgment of the DSMB and reflect all information available to the DSMB at the time the recommendations are made.

III. Reporting of Adverse and Serious Adverse Events

The intervention is non-invasive and inherently low-risk. Therefore, adverse events (AEs) are likely to be do to life events other than study participation and the intervention. Note that in our prior R21, no AEs occurred that were deemed related to the study protocol.

All documentation of AEs and other reportable events will be shared with the RAND institutional review board (IRB), i.e., the RAND Human Subjects Protections Committee (HSPC), the Makerere University IRB, the DSMB, and the NIH Program Official in writing, in accordance with the time schedule specified by NIH. Specifically, any unexpected, serious adverse events that occur during the course of this investigation will be reported by Drs. Wagner or Wanyenze within the next business day to the study IRBs via phone, email, and/or a message in the RAND HSPC's web portal. This report will be followed by written report within 3 business days to the IRB, and within 10 business days to the NIH Program Official. The written report will contain: Identifying information for the research protocol (e.g., project title, investigator's name, and the grant/contract number); date on which the event occurred and the date at which the PI became aware of the event; detailed description of the event and impact on the participant(s); detailed description of the measures taken (including clinical) in response to the event (if any); confirmation that the appropriate monitoring entities and regulatory bodies have been notified as needed; and a description of any changes to the protocol or other corrective actions that have been taken or are proposed in response to the event. In consultation with the IRBs, the DSMB, and NIH, Wagner, Bogart and Wanyenze, will address whether there is a need to redesign or amend the protocol, and/or to inform current and future subjects of a change in description of risk (e.g., in consent form and protocol).

IV. Assessment and Appropriate Action Regarding Risk of Harm to Self or Others

We have established procedures and guidelines to respond to risk disclosures and crisis situations among study participants, which we used in our prior R21. Intervention and interview staff will be trained to recognize risks or crises that require the implementation of the following protocol. The MPIs will be responsible for the reporting of any adverse events or social harms to the IRBs and NIH. Adverse events and social harms will be reported within in compliance with the RAND HSPC reporting policies and will also be reported to the NIH Program Official in writing, in accordance with the time schedule specified by NIH.

Suicidal Ideation or Intention to Harm Self: There is a risk that a participant will disclose intentions to kill themselves or do serious harm to self. If there are indications during the survey or intervention sessions that the participant poses a risk of suicide or doing harm to him/herself, the interviewer or facilitator will immediately contact the Project Director (Gwokyalya), who will then inform Drs. Wanyenze and Wagner. The interviewer or facilitator should assess the participant for safety (immediate suicidal plan), as noted below, and given referrals for further evaluation and care to their counselor at the study clinic:

- Before the participant leaves, the interviewer should explain to the participant that they would like an on-site mental health counselor to speak with him/her.
- The interviewer would call the on-site mental health team counselor at the clinic, who would then conduct an assessment of the risk for potential harm and the appropriate action in terms of evaluating the participant's need for mental health services and notifying appropriate authorities. This assessment would be done before the participant leaves the premises.

If a mental health counselor is not available to make an evaluation at that time, the interviewer or facilitator will make the assessment by assessing the nature and severity of the risk that the participant poses to him/herself. The interviewer will ask:

- If s/he has taken any active steps to carry out the harm (e.g., took pills, acquired a weapon);
- If s/he has a plan: If the participant has a plan, the interviewer will explore the nature of the plan and the level of imminent risk for carrying out the plan; and
- History of having harmed self (past attempts).

Based on these criteria, the interviewer or intervention staff will determine if the situation is an emergency, non-

emergency, or uncertain and requiring further consultation with the Project Director and with a counselor at the clinic, when one is available. Interviews will be conducted at the clinic sites during clinic business hours and thus on-site healthcare professionals will be available with whom to consult.

Emergency: In the rare instance that a participant is in imminent danger and needs emergency medical or mental health services (thinking of harming themselves and has a plan to do so), the intervention or interview staff will call the authorities. The interviewer will then immediately call the Project Director (Gwokyalya) and write an incident report within 1 business day (i.e., an email to Drs. Wagner and Wanyenze with the participant ID and a summary of the assessment and action taken). The PIs will inform the IRBs.

Non-emergency: If a participant spontaneously reports that s/he has had passing thoughts of suicide, but on further questioning clearly states that s/he has no intention of plan for self-harm, the interviewer will provide appropriate referrals to local mental health resources, including information about the clinic's mental health counselors, and answer any questions the participant may have about these services.

If the study team member is uncertain as to the severity of the risk: S/he will call the Project Director immediately and discuss the incident prior to the participant leaving the premises. The Project Director will determine whether further action is needed and file a serious adverse event report, if necessary. The interviewer will also provide the participant with a written list of mental health referral numbers.

Intent to Harm Others or Child or Elder Abuse/Neglect. If a statement is made during any project session about intent to harm others, the staff member will follow similar procedures as listed above under "Suicidal Ideation or Intent to Harm Self." If child or elder abuse or neglect is disclosed, the staff will follow the procedures discussed above for assessment and reporting.

V. Stopping Rules for Clinical Trial

If there is clear evidence of harm, we will stop the clinical trial. Although we do not expect any physical harm or serious psychological harm beyond minimal distress, we have procedures in place for monitoring harm from the intervention, including asking participants to contact us if they experience any adverse events, offering additional resources to those with very high levels of mental health symptoms that we identify through data monitoring, and providing resources after baseline and at every follow-up time point.

VI. Data Safeguarding Plan

<u>i. Project Leadership</u>

The Principal Investigators are Drs. Glenn Wagner and Laura Bogart.

<u>ii. Project Description and Types of Data</u>

A. Project Description.

The goals of this project are to:

1. Conduct a multisite RCT of the GC-CCP network-based advocacy strategy to evaluate effects on CC screening uptake among unscreened alters across urban/rural and public/private clinics.
2. Use a mixed methods approach (semi-structured interviews and administrative clinic data) to examine clinic-, provider-, and client-level barriers and facilitators of GC-CCP Implementation and Sustainment.
3. Examine mediators and moderators (among index, alter and network characteristics) of intervention effects on (a) alter CC screening; and (b) engagement in CC prevention advocacy of index and alter (1st and 2nd degree) to better understand its multiplier effect on diffusion of advocacy throughout a network.
4. Evaluate the cost-effectiveness of Implementing GC-CCP to increase CC screening and low cost, early-stage treatment, and prevent advanced disease, compared to enhanced usual care.

B. Types of Data.

Medical chart data: We will obtain date related to cervical cancer (CC) screening and treatment services, including biopsies, received for all index and alter participants.

Survey and social network assessment data: The survey assessments will be administered using Network Canvas, and will collect information about participant demographics; use of CC screening and treatment services; engagement in CC prevention advocacy; CC knowledge/beliefs; self-efficacy related to use of CC-related medical services and engagement in CC prevention advocacy; sharing of personal experience related to CC screening; CC internalized and enacted stigma; social support; and relationship status and sexual

behavior. During the social network assessment portion, participants will be asked to list 10 adult females in their social network (e.g., friends, family, other community members). We will collect first name and last initial for each social network member to avoid the potential for confusing individuals who might have the same first and last initial, and to assist with linking network member data across assessments (to assess network change over time). For each social network member (alter) listed we will collect information about demographics; description of the relationship between the index participant and the individual alter; CC prevention advocacy provided to the alter by the index participant; social support provided by the alter; and the alter's expression of CC-related stigma. We will also ask for a description of how often individuals listed in the social network assessment interact with each other.

Individual qualitative interview and focus group discussion data. Individual qualitative interviews will be conducted with providers involved with providing CC-related services, clinic administrators, and ministry of health policy makers; these interviews will focus on the respondent's perspective of CC-related services provided at the clinic, barriers to good access to CC screening and ability to scale-up provision of CC screening, and views on how to optimize client engagement in the GC-CCP intervention. Female clients of the clinics will participate in focus group discussions to share their views on CC-related services provided at the clinic, barriers to good access to CC screening, and views on how to optimize client engagement in the GC-CCP intervention.

Fidelity ratings by facilitators and supervisors. These are ratings of session content covered, participant engagement, and challenges incurred, during each completed intervention session.

<u>iii. Data Sensitivity</u>

Data transferred to RAND will not contain direct identifiers. Patient participant survey data will contain the first name and last name initial of social network members elicited in their survey. This level of detail and identifiability is necessary to be able to assess network connectivity and overlap across different social networks.

A. Types of Identifiers

   1. Participant identifying data

Identifying information for client participants will consist of name, birth date, neighborhood address, telephone number, and contact information of close family or friend to be used to facilitate maintaining contact with the participant through the course of their study participation. Provider participants will provide name and phone number. These data will only be available under lock and key at the clinical site where the patient is seen and will never be in any electronic dataset or case report form.

To the extent legally possible, we intend to maintain the confidentiality of this information as it pertains to the identity of participants. We will not include names of participants in reports resulting from this study.

B. Raw Data in Need of Data Safeguarding

   The following are the items to be safeguarded:

   1. Signed written consent form will contain participant name and ID number.
   2. Contact information Forms

The contact information forms of enrolled participants that contain the participants' names, address and phone number, other contact information and ID numbers. This information will be kept under lock and key at the study site. This data will not be entered into any database.

   3. Worksheets and Logs

Worksheets used by field staff and operations staff that contain the participant name and ID link. This information will be kept under lock and key at the study site. This data will not be entered into any database.

   4. Digital recordings of individual interviews and focus groups

These recordings will include no identifying information and recordings will be deleted from the recording devise once uploaded to the computer, and then deleted from the computer with Eraser software once a transcript has been completed. The transcript file from the interviews will be saved in an encrypted folder on the password protected study computer.

C. Computerized Data in Need of Data Safeguarding

There will be no electronic file which links the primary data identifiers to assigned ID numbers.

All survey data is collected with the computer-assisted software and a tablet. The survey data is stored in the password protected study computer and in an encrypted folder on that computer.

Medical records data will be abstracted from medical charts by study coordinators and recorded in excel spreadsheets, with data linked to participant only via study ID number (no identifying information will be on the spreadsheet).

iv. Data Safeguarding and Transmittal Procedures

MakSPH will be responsible for safely storing data prior to its transmittal to RAND. MakSPH Project Director will retain a matching file that links study IDs to participant names for patients enrolled in the RCT who need to be followed over time. The matching file will be password protected and stored on a password protected study computer. To protect confidentiality, all hard copy research data will be kept in locked file cabinets and will be available only to members of the study team. All study devices used to collect or store data will use the manufacturer-supplied encryption of the device's entire storage area, will require a password at log in, and available to study staff only. All digital files will be stored on password protected computers and MakSPH servers accessible to study staff only. MakSPH will remove identifying information from datasets prior to transmittal to RAND (except the first name and last initial of social network members as noted below). Participant names will be replaced with a study ID during this process to facilitate data matching at RAND. RAND staff will review data files upon receipt to ensure that deidentification has been performed before moving to a RAND server.

After completion of individual qualitative interviews of focus group discussions (FGD), the interviewer or facilitator will upload the digital recording to the study computer (at the study office) and save the file in an encrypted folder. This will be done within 24 hours of the interview/FGD being completed. Once the file has been uploaded to the computer, the file on the recording device will be immediately deleted. The interviewer will create a transcript of the recording within one week of the interview being completed, after which the computer file of the recording will be deleted using Eraser software to secure the deletion. The audio and transcript files will contain no identifying information.

RAND will receive data from surveys, social network assessments, qualitative interview and focus group transcripts, fidelity ratings, and medical record data. Patient participant social network assessment data will include the first name and last initial of their listed social network members. Cervical cancer data will not appear along with any personal identifying information. Medical records data will be identifiable only by study ID numbers. Participant workshop evaluation forms will be entered into an Excel spreadsheet by study staff and transferred to RAND via Kiteworks. The evaluations will be identified by study ID in the file transmitted to RAND.

Survey data can be exported from the Network Canvas Interviewer module directly to Excel, such that the Server module is not needed. The study interviewers will export survey data directly to Excel and .graphml files (Network Canvas specific file type, that can only be opened with NC software). Each data collector exports his/her survey data collected and uploads it directly to Kiteworks. If the Server module becomes necessary for field staff, the data transfer from individual devices to the central study computer would occur over a secure network. Per the Network Canvas website (https://documentation.networkcanvas.com/reference/irb-best-practices/), "Our remote transfer functionality (i.e., the workflow with Server) uses end-to-end encryption to ensure that your data cannot be intercepted by a third party as it is sent remotely back to your laboratory or department computer. This transfer can take place on a local network within your institution, for added security. Data transmitted between Network Canvas and Server are encrypted using a symmetric encryption algorithm called Advanced Encryption Standard (AES) which secures its integrity before transmission. Once the data are encrypted, they are sent securely to Server using Transport Layer Security (TLS)." Further, "Server is a desktop application that facilitates secure transfer and management of study data, but it does not provide a storage solution. We do not transmit, collect or retain any data from or about any study. The data collected in the field is yours, and is only ever stored on your devices. Additionally, we do not use cookies or other tracking tokens of any kind within Network Canvas." RAND staff may utilize Server to merge individual survey data files transmitted via Kiteworks. This will be done on a RAND supplied device on a secure network.

Study data will be transmitted to RAND via Kiteworks, RAND's secure file-sharing website. The Kiteworks project workspace will be password-protected and only accessible by authorized project staff. Data sent to RAND via Kiteworks is encrypted during transmission and encrypted at rest. Once files are uploaded to Kiteworks, members of the RAND team responsible for maintaining the site will download the files, ensure deidentification has been performed, and then upload to a secure RAND server that can be accessed only by authorized personnel.

Personal information including participants' name, address, and phone number will be stored separately from all research data by MakSPH. All data collected will be kept confidential and not shared with the patient

participant's physician or other clinic staff, or any of their social network members whom they may recruit to participate. Participants will be informed of these conditions during the informed consent process.
Study staff at Indiana University will receive fully de-identified data only from RAND via Kiteworks.
Data will not be shared with individuals who are not part of the project team. All study staff will receive training on confidentiality.

<u>v. Responsibility for Data Safeguarding</u>
Dr. Wagner has ultimate responsibility for data safeguarding. Violet Gwokyalya (Project Director from Makerere University School of Public Health (MakSPH)) will be responsible for day-to-day oversight of the data collection and data management, and for oversight of all staff who handle participant-identifying computer files and other identifiable data.

<u>vi. Disclosure Risks</u>
This project will collect sensitive information about participants, including diagnosis and history of cervical cancer diagnosis or risk factors, HIV status, and sexual behavior. Because of stigma attached these conditions and behaviors, disclosure—whether accidental or not—has risks related to social stigma and shame, abandonment and abuse, as well as potential benefits in the form of social support.

<u>vii. Audit and Monitoring Plans</u>
Dr. Wagner will follow up with project staff at periodic intervals to ensure that they are in compliance with the data safeguarding procedures described in Section V.

<u>viii. Disposition of Data after the Study</u>
Hard and soft copies of raw data, including participant identifiable information will be kept at MakSPH, and files with de-identified data will be kept at all participating institutions. All linking files and identifiable information will be destroyed within a year of study completion. Signed consent forms will be retained by MakSPH for a period of 5 years, per Makerere University Research & Ethics Committee protocol.
Following publication of the main study papers, de-identified survey data will be stored in a database and made publicly available to researchers who successfully complete a registration process after requesting to use the data. The data made available through this process will not contain any direct or indirect identifiers. Users must agree to the conditions of use governing access to the public release data, including restrictions against attempting to identify study participants, destruction of the data after analyses are completed, reporting responsibilities, restrictions on redistribution of the data to third parties, and proper acknowledgement of the data resource. Users must notify the institutional review board of their institution of their intention to use the data and their procedures for data management and security. Users must submit proposals regarding intended use of the data; the RAND study team will determine the scientific soundness of the proposal as part of the decision for the researcher to be able to access the dataset.
<u>ix. Employee Education about Confidentiality</u>
All study personnel who will be handling any participant-identifying worksheets, or medical record information as well as the survey interviewers and their supervisor will sign an oath of confidentiality. As part of their training, they will be instructed in the rules of confidentiality and data safeguarding for the study.

G. STUDY TIMELINES

**Activity**	**Year 1 (2023/24)**				**Year 2 (2024/25)**				**Year 3 (2025/26)**				**Year 4 (2026/27)**				**Year 5 (2027/28)**			
<u>Preparation.</u> Interviews with facility administration MoH and providers to identify modifiable clinic and provider level barriers to CC screening	■																			
Data analysis of barriers and gaps		■																		
Develop standardized provider training manual		■	■																	

Provider training on the importance of recommending CC screening			■																	
Implementation of quality improvement approaches				■																
Implementation. Recruitment and baseline surveys interviews for index and 1st degree alters				■																
Conduct a Peer-facilitated advocacy training					■															
Follow-up assessments (index and 1st degree alters)							■	■			■	■								
Recruitment and baseline surveys interviews for 2nd degree alters								■												
Follow-up assessments (2nd degree alters)										■	■		■	■						
Chart abstraction					■	■	■	■	■	■										
Data collection on cost effectiveness					■	■	■	■	■	■	■									
Implement GC-CCP with clients not enrolled in the trial											■	■	■	■	■	■	■	■	■	■
Statistical analysis (including cost effectiveness analysis)												■	■	■	■	■	■	■	■	■
Results dissemination																			■	■

References

1. Ferlay J, Ervik M, Lam F, Colombet M, Mery L, Piñeros M, Znaor A, Soerjomataram I, Bray F (2018). Global Cancer Observatory: Cancer Today. Lyon, France: International Agency for Research on Cancer.

2. African Cancer Registry Network: Kampala Cancer Registry. 2018.

3. Wabinga HR, Parkin DM, Nambooze S. Kampala Cancer Registry Report for the Period 2007-2009. Kampala, Uganda: Kampala Cancer Registry, 2012.

4. International Agency of Cancer Registries: Kampala Cancer Registry Profile Page. Lyon: International Agency for Research on Cancer (IARC); 2018.

5. Nakisige C, Schwartz M, Ndira AO. Cervical cancer screening and treatment in Uganda. *Gynecologic oncology reports* 2017; 20: 37-40.

6. Nooh AM, Mohamed Mel-S, El-Alfy Y. Visual Inspection of Cervix With Acetic Acid as a Screening Modality for Cervical Cancer. J Low Genit Tract Dis. 2015 Oct;19(4):340-4.

7. WHO guidelines for the use of thermal ablation for cervical pre-cancer lesions. Geneva: World Health Organization; 2019. Executive summary.

8. Ndejjo R, Mukama T, Musabyimana A, Musoke D. Uptake of Cervical Cancer Screening and Associated Factors among Women in Rural Uganda: A Cross Sectional Study. *PloS one* 2016; 11(2): e0149696.

9. Bruni L, Albero G, Serrano B, Mena M, Gómez D, Muñoz J, Bosch FX, de Sanjosé S. ICO/IARC Information Centre on HPV and Cancer (HPV Information Centre). Human Papillomavirus and Related Diseases in Uganda. Summary Report 17 June 2019.

10. Union for International Cancer Control (UICC). Cervical cancer elimination in Africa: where are we now and where do we need to be? April 2022.

11. Ugandan Ministry of Health. Strategic plan for cervical cancer prevention and control in Uganda 2010–2014. April, 2010. Kampala.

12.. Black E, Hyslop F, Richmond R. Barriers and facilitators to uptake of cervical cancer screening among women in Uganda: a systematic review. *BMC Women's Health.* 2019;19:108.

13. Teng FF, Mitchell SM, Sekikubo M, et al. Understanding the role of embarrassment in gynaecological screening: a qualitative study from the ASPIRE cervical cancer screening project in Uganda. BMJ Open 2014;4:e004783.

14. Ndejjo R, Mukama T, Musabyimana A, Musoke D. Uptake of Cervical Cancer Screening and Associated Factors among Women in Rural Uganda: A Cross Sectional Study. *PLoS One*. 2016;11(2):e0149696.

15. Paul P, Winkler JL, Bartolini RM, Penny ME, Huong TT, Nga LT, Kumakech E, Mugisha E, Jeronimo J. Screen-and-Treat Approach to Cervical Cancer Prevention Using Visual Inspection With Acetic Acid and Cryotherapy: Experiences, Perceptions, and Beliefs from Demonstration Projects in Peru, Uganda, and Vietnam. *The Oncologist*. 2013;18:1278-1284.

16. Ndejjo R, Mukama T, Kiguli J, . Knowledge, facilitators and barriers to cervical cancer screening among women in Uganda: a qualitative study. *BMJ Open*. 2017;7:e016282.

17. Medley A, Kennedy C, O'Reilly K, Sweat M. Effectiveness of peer education interventions for HIV prevention in developing countries: a systematic review and meta-analysis. AIDS Educ Prev 2009; 21; 181-206.

18. Maiorana, A., Kegeles, S., Fernandez, P., Salazar, X., Caceres, C., Sandoval, C., Rosasco, A. M. & Coates, T. (2007) Implementation and evaluation of an HIV/STD intervention in Peru. *Evaluation and Program Planning,* 30(1), pp. 82-93.

19. Rogers, E.M. (1983). Diffusion of innovations (2nd edition). New York: Free Press.

20. Broadhead RS, et al. Harnessing peer networks as an instrument for AIDS prevention: Results from a peer driven intervention. Public Health Reports 1998; 113: 42-7.

21. Kelly, J. A. (2004) Popular opinion leaders and HIV prevention peer education: resolving discrepant findings, and implications for the development of effective community programmes. *AIDS Care,* 16(2), pp. 139-50.

22. Bell DC, Montoya ID, Atkinson JS, Yang S-J. Social networks and forecasting the spread of HIV infection. J Acquir Immune Defic Syndr 2002; 31:218-229.

23. Latkin CA, Sherman S, Knowlton A. HIV prevention among drug users: Outcome of a network-oriented peer outreach intervention. Health Psychology 2003; 22:332-9.

24. Sikkema K.J., Kelly J.A., Winett R.A., Solomon L.J., Cargill V.A., Roffman R.A., et al. Outcomes of a randomized community level HIV prevention intervention for women living in 18 low income housing developments. Am J Public Health 2000;90:57–63.

25. Friedman SR, Maslow C, Bolyard M, et al. Urging others to be healthy: "Intravention" by injection drug users as a community prevention goal. AIDS Education and Prevention 2004; 16:250-63.

26. Li M, Nyabigambo A, Navvuga P, Nuwamanya E, Nuwasiima A, Kaganda P, Asiimwe FT, Vodicka E, Mugisha NM, Mukose A, Kwesiga DK, Lubinga SJ, Garrison LP, Babigumira JB. Acceptability of cervical cancer screening using visual inspection among women attending a childhood immunization clinic in Uganda. Papillomavirus Res. 2017; 4:17-21.

27. Mutyaba, T, Mirembe, F, Sandin, S and Weiderpass, E. Male partner involvement in reducing loss to follow-up after cervical cancer screening in Uganda. *International Journal of Gynecology & Obstetrics*. 2009;107:103-106.

28. Bogart LM, Matovu JKB, Wagner GJ, Green HD, Storholm ED, Klein DJ, Marsh T, MacCarthy S, Kambugu A. A Pilot Test of Game Changers, a Social Network Intervention to Empower People with HIV to be Prevention Advocates in Uganda. AIDS Behav. 2020 Sep;24(9):2490-2508.

29. Christakis, Nicholas A., and James H. Fowler. "The collective dynamics of smoking in a large social network." *New England journal of medicine* 358, no. 21 (2008): 2249-2258.

30. Go Myong-Hyun, Joan S. Tucker, Harold D. Green Jr, Michael Pollard, and David Kennedy. "Social distance and homophily in adolescent smoking initiation." *Drug and alcohol dependence* 124, no. 3 (2012): 347-354.

31. Roura M., Urassa M., Busza J., Mbata D., Wringe A., Zaba B.Scaling Up Stigma? The effects of antiretroviral roll-out on stigma and HIV testing. Early evidence from rural Tanzania. Sexually Transmitted Infections 2009; 85:308-312.

32. Ministry of Health (MOH) [Uganda} and ORC Macro. 2006. *Uganda HIV/AIDS Sero-Behavioral Survey 2004-2005.* Calverton, Maryland, USA: Ministry of Health and ORC Macro.

33. Nam, S.L., et al. (2008) The relationship of acceptance or denial of HIV-status to antiretroviral adherence among adult HIV patients in urban Botswana. Soc Sci Med, 67(2) p. 301-10

34. Wagner GJ, Ryan G, Huynh A, Kityo C & Mugyenyi P. A qualitative analysis of the economic impact of HIV and ART on individuals and households in Uganda. *AIDS Patient Care and STDs* 2009; 23:793-8.

35. Kegeles, S.M., Hays, R.B., Pollack, L.M., and Coates, T.J., *Mobilizing young gay and bisexual men for HIV prevention: a two-community study.* AIDS, 1999. 13(13): p. 1753-62.

36. Kelly, J. A. (2004) Popular opinion leaders and HIV prevention peer education: resolving discrepant findings, and implications for the development of effective community programmes. *AIDS Care,* 16(2), pp. 139-50.

37. Medley A, Kennedy C, O'Reilly K, Sweat M. Effectiveness of peer education interventions for HIV prevention in developing countries: a systematic review and meta-analysis. AIDS Educ Prev 2009; 21; 181-206.

38. Maiorana, A., Kegeles, S., Fernandez, P., Salazar, X., Caceres, C., Sandoval, C., Rosasco, A. M. & Coates, T. (2007) Implementation and evaluation of an HIV/STD intervention in Peru. *Evaluation and Program Planning,* 30(1), pp. 82-93.

39. Aarons, G. A., Hurlburt, M., & Horwitz, S. M. (2011). Advancing a conceptual model of evidence-based practice implementation in public service sectors. *Adm Policy Ment Health*, *38*(1), 4-23.

40. Moullin, J. C., Dickson, K. S., Stadnick, N. A., Rabin, B., & Aarons, G. A. (2019). Systematic review of the Exploration, Preparation, Implementation, Sustainment (EPIS) framework. *Implementation Science*, *14*(1), 1.

41. Wanyenze RK, Bwanika JB, Beyeza-Kashesya J, Mugerwa S, Arinaitwe J, Matovu JKB, Gwokyalya V, Kasozi D, Bukenya J, Makumbi F. Uptake and correlates of cervical cancer screening among HIV-infected women attending HIV care in Uganda. Glob Health Action. 2017;10(1):1380361.

42. Bukirwa A, Mutyoba JN, Mukasa BN, Karamagi Y, Odiit M, Kawuma E, Wanyenze RK. Motivations and barriers to cervical cancer screening among HIV infected women in HIV care: a qualitative study. BMC Womens Health. 2015 Oct 12;15:82.

43. Matovu JKB, Bogart LM, Nakabugo J, Kagaayi J, Serwadda D, Wanyenze RK, Ko AI, Kurth AE. Feasibility and acceptability of a pilot, peer-led HIV self-testing intervention in a hyperendemic fishing community in rural Uganda. PLoS One. 2020 Aug 7;15(8):e0236141.

44. Matovu JKB, Nambuusi A, Nakabirye S, Wanyenze RK, Serwadda D. Formative research to inform the development of a peer-led HIV self-testing intervention to improve HIV testing uptake and linkage to HIV care among adolescents, young people and adult men in Kasensero fishing community, Rakai, Uganda: a qualitative study. BMC Public Health. 2020 Oct 20;20(1):1582.

45. Matovu JKB, Kisa R, Buregyeya E, Chemusto H, Mugerwa S, Musoke W, Vrana CJ, Malek AM, Korte JE, Wanyenze RK. 'If I had not taken it [HIVST kit] home, my husband would not have come to the facility to test for HIV': HIV self-testing perceptions, delivery strategies, and post-test experiences among pregnant women and their male partners in Central Uganda. Glob Health Action. 2018;11(1):1503784.

46. Wagner GJ, Matovu JKB, Juncker M, Namisango E, Bouskill K, Nakami S, Beyeza-Kashesya J, Luyirika E, Bogart LM, Grenn HD, Wanyenze RK. Effects of a group advocacy intervention on cervical cancer screening among social network members: results of a randomized controlled trial in Uganda. Under review.

47. Wagner, Glenn J., Laura M. Bogart, Harold D. Green, Erik D. Storholm, David J. Klein, Ryan K. McBain, Richard Serunkuuma, Kuraish Mubiru, Joseph KB Matovu, and Stephen Okoboi. "Social network-based group intervention to promote HIV prevention in Uganda: study protocol for a cluster randomized controlled trial of Game Changers." *Trials* 23, no. 1 (2022): 1-10.

48. Bogart, L. M., Wagner, G. J., Musoke, W., Naigino, R, Linnemayr, S., Maistrellis, E., Klein, D. J., Jumamil, R. B., Mukasa, B., & Bassett, I. V. (2017). A comparison of home-based versus outreach event-based community HIV testing in Ugandan fisherfolk communities. AIDS and Behavior, 21(2), 547-560.

49. Bogart L.M., Musoke W., Mayatsa J, Marsh T., Naigino R., Banegura A., Mukama C.S., Allupo S., Odiit M., Kadama H., Mukasa B., Wanyenze RK. Recommendations for improving oral pre-exposure prophylaxis (PrEP) Implementation and social marketing in Ugandan fisherfolk communities: A qualitative exploration. In Press. Community Health Equity Research and Policy.

50. Ober AJ, Watkins KE, Hunter SB, Ewing B, Lamp K, Lind M, Becker K, Heinzerling K, Osilla KC, Diamant AL, Setodji CM. Assessing and improving organizational readiness to implement substance use disorder treatment in primary care: findings from the SUMMIT study. BMC Fam Pract. 2017 Dec 21;18(1):107.

51. Ober AJ, Watkins KE, McCullough CM, Setodji CM, Osilla K, Hunter SB. Patient predictors of substance use disorder treatment initiation in primary care. J Subst Abuse Treat. 2018 Jul;90:64-72.

52. Ober AJ, Murray-Krezan C, Page K, Friedmann PD, Chan Osilla K, Ryzewicz S, Huerta S, Mazer MW, Leamon I, Messineo G, Watkins KE, Nuckols T, Danovitch I. The Substance Use Treatment and Recovery Team (START) study: protocol for a multi-site randomized controlled trial evaluating an intervention to improve initiation of medication and linkage to post-discharge care for hospitalized patients with opioid use disorder. Addict Sci Clin Pract. 2022 Jul 28;17(1):39.

53. Ober AJ, Takada S, Zajdman D, Todd I, Horwich T, Anderson A, Wali S, Ladapo JA. Factors affecting statin uptake among people living with HIV: primary care provider perspectives. BMC Fam Pract. 2021 Oct 30;22(1):215.

54. Green HD, Hoover MA, Wagner GJ, Ryan GW, Ssegujja E. Measuring agreement between egos and alters: understanding informant accuracy in personal network studies. Field Methods 2014; 26:126-140.

55. Johnson, Laura M., Harold D. Green, Minggen Lu, Jamila K. Stockman, Marisa Felsher, Alexis M. Roth, and Karla D. Wagner. "Who Can I Ask? Who Would I Tell? An Egocentric Network Analysis Among a Sample of Women At-Risk to Explore Anticipated Advice Seeking and Disclosure Around Pre-exposure Prophylaxis (PrEP)." *AIDS and Behavior* (2022): 1-15.

56. Tumwine C, Nannungi A, Ssegujja E, et al. An exploratory study of HIV-prevention advocacy by persons in HIV care in Uganda. *African Journal of AIDS Research.* 2011;10(4):427-433.

57. Tumwine C, Mbona Tumwesigye N, Mugyenyi P, Wagner G. Psychosocial Characterization Of HIV Clients With Potential To Be Change Agents For HIV Prevention In Uganda. *Journal of Clinical Research in HIV AIDS and Prevention.* 2012;1(1):3-11.

58. Wagner GJ, Ghosh-Dastidar B, Slaughter ME. Engagement in HIV prevention advocacy associated with increased consistent condom use among HIV clients in Uganda. *AIDS and Behavior.* 2015;19(7):1150-1156.

59. Ssali S, Wagner G, Tumwine C, Nannungi A, Green H. HIV clients as agents for prevention: a social network solution. *AIDS research and treatment.* 2012;2012.

60. Wanyenze RK, Matovu JKB, Bouskill K, Juncker M, Namisango E, Nakami S, Beyeza-Kashesya J, Luyirika E, Wagner GJ. Social network-based group intervention to promote uptake of cervical cancer screening in Uganda: study protocol for a pilot randomized controlled trial. *Pilot and Feasibility Studies*. In press.

61. Powell BJ, Waltz TJ, Chinman MJ, Damschroder LJ, Smith JL, Matthieu MM, Proctor EK, Kirchner JE. A refined compilation of implementation strategies: results from the Expert Recommendations for Implementing Change (ERIC) project. Implement Sci. 2015 Feb 12;10:21.

62. Glasgow RE, Vogt TM, Boles SM. Evaluating the public health impact of health promotion interventions: the RE-AIM framework. American journal of public health. 1999 Sep;89(9):1322-7

63. Deming, W., *Out of the crisis, 1986*. Vol. 507. 1991, Cambridge, MA: Massachusetts Institute of Technology Center for Advanced Engineering Study iii.

64. Berwick, D.M., *Developing and testing changes in delivery of care.* Ann Intern Med, 1998. 128(8): p. 651-6.

65. Chinman, M., S. Hunter, and P. Ebener, *Employing continuous quality improvement in community-based substance abuse programs.* International Journal of Health Care Quality Assurance, 2012. **25**(7): p. 606-617.

66. Hunter, S.B., et al., *Promoting Success: A Getting to Outcomes guide to implementing continuous quality improvement for community service organizations*2015, RAND Corporation.

67. Hennink M, Kaiser BN. Sample sizes for saturation in qualitative research: A systematic review of empirical tests. Social Science & Medicine. 2022 Jan 1;292:114523.

68. Proctor E, Silmere H, Raghavan R, Hovmand P, Aarons G, Bunger A, et al. Outcomes for implementation research: conceptual distinctions, measurement challenges, and research agenda. Adm Policy Ment Health. 2011;38(2):65-76.

69. Bernard H. Research Methods in Anthropology: Qualitative and Quantitative Approaches. 4th ed. Lanham, MD: AltaMira Press; 2006.

70. Jehn KA, Doucet L. Developing categories for interview data: consequences of different coding and analysis strategies in understanding text, part 2. *Field Methods*. 1997;9(1):1-7.

71. Ryan GW, Bernard HR. Data management and analysis methods. In: Denzin N, Lincoln Y, editors. Handbook of Qualitative Research. 2nd ed. Thousand Oaks: Sage Publications; 2000. p. 769-802.

72. Cohen J. A Coefficient of agreement for nominal scales. *Educational and Psychological Measurement*. 1960;20(1):37-46.

73. Damschroder, L.J., et al., *Fostering implementation of health services research findings into practice: a consolidated framework for advancing implementation science.* Implement Sci, 2009. 4: p. 50.

74. Matovu JK, Wanyenze RK, Mawemuko S, Okui O, Bazeyo W, Serwadda D. Strengthening health workforce capacity through work-based training. BMC Int Health Hum Rights. 2013;13:8

75. Curran, G. M., Bauer, M., Mittman, B., Pyne, J. M., & Stetler, C. (2012). Effectiveness-implementation hybrid designs: combining elements of clinical effectiveness and implementation research to enhance public health impact. *Med Care*, *50*(3), 217-226.

76. Festinger, L. (1957). *A Theory of Cognitive Dissonance*. Stanford, CA: Stanford University Press.

77. Uganda Ministry of Health. Annual Health Sector Performance Report 2020/2021.

78. Torgerson DJ. Contamination in trials: is cluster randomisation the answer? *BMJ.* 2001;322(7282):355-7.

79. Kalichman SC, Simbayi LC, Jooste S, et al. Development of a brief scale to measure AIDS-related stigma in South Africa. *AIDS Behav.* 2005;9(2):135-143.

80. Marlow LA, Wardle J. Development of a scale to assess cancer stigma in the non-patient population. BMC Cancer. 2014 Apr 23;14:285.

81. Cho J, Smith K, Choi EK, Kim IR, Chang YJ, Park HY, Guallar E, Shim YM. Public attitudes toward cancer and cancer patients: a national survey in Korea. Psychooncology. 2013 Mar;22(3):605-13.

82. Moser A, Stuck AE, Silliman RA, Ganz PA, Clough-Gorr KM. The eight-item modified Medical Outcomes Study Social Support Survey: psychometric evaluation showed excellent performance. Journal of clinical epidemiology. 2012 Oct 1;65(10):1107-16.

83. McCarty C, Bernard H, Killworth P, Shelley GA, Johnsen EC. Eliciting representative samples of personal networks. *Soc Networks.* 1997;19:303-323.

84. Wagner GJ, Ghosh-Dastidar B, Garnett J, Kityo C, Mugyenyi P. Impact of HIV antiretroviral therapy on depression and mental health among clients with HIV in Uganda. *Psychosomatic Medicine* 2012; 74:883-90.

85. Wagner GJ, Linnemayr S, Goggin K, Mindry D, Beyeza-Kashesya J, Finocchario-Kessler S, Robinson E, Birungi J, Wanyenze RK. Prevalence and correlates of use of safer conception methods in a cohort of Ugandan HIV-affected couples with fertility intentions. AIDS & Behavior. 2017; 21(8):2479-2487.

86. Hedges LV, Citkowicz M. Estimating effect size when there is clustering in one treatment group. *Behav Res Methods,*. 2015;47(4):1295-308.

87. Perry BL, Pescosolido BA, Borgatti SP. Egocentric network analysis: foundations, methods, and models: Cambridge University Press; 2018.

88. enny DA, Kashy DA. Dyadic data analysis using multilevel modeling. Handbook of advanced multilevel analysis: Routledge; 2011. p. 343-78.

89. Kenny DA, Kashy DA, Cook WL. Dyadic data analysis: Guilford press; 2006.

90. Huber PJ, editor. The behavior of maximum likelihood estimates under nonstandard conditions. Proceedings of the fifth Berkeley symposium on mathematical statistics and probability; 1967: University of California Press.

91. Wooldridge JM. Econometric analysis of cross section and panel data: MIT press; 2010.

92. Turner EL, Prague M, Gallis JA, Li F, Murray DM. Review of recent methodological developments in group-randomized trials: part 2—analysis. *Am J Public Health,*. 2017;107(7):1078-86.

93. Baldwin SA, Bauer DJ, Stice E, Rohde P. Evaluating models for partially clustered designs. *Psychol Methods,*. 2011;16(2):149.

94. Aiken LS, West SG. Multiple regression: testing and interpreting interactions. Thousand Oaks, CA: Sage; 1991 1991.

95. Baron RM, Kenny DA. The moderator-mediator variable distinction in social psychological research: conceptual, strategic, and statistical considerations. *J Pers Soc Psychol*. 1986; 51(6):1173-82.

96. Preacher KJ, Hayes AF. SPSS and SAS procedures for estimating indirect effects in simple mediation models. *Behav Res Methods Instrum Comput,*. 2004;36(4):717-31.

97. Preacher KJ, Hayes AF. Asymptotic and resampling strategies for assessing and comparing indirect effects in multiple mediator models. *Behav Res Methods,*. 2008;40(3):879-91.

98. Cost-effectiveness in health and medicine. New York, NY: Oxford University Press; 1996.

99. Campbell MK, Torgerson DJ. Bootstrapping: estimating confidence intervals for cost-effectiveness ratios. *QJM*. 1999;92(3):177-82.

100. Gorsky RD. A method to measure the costs of counseling for HIV prevention. *Public Health Rep,*. 1996;111(Suppl 1):115.

101. Crease A, Parker D. Cost analysis in primary health care. *Geneva, Switzerland: World Health Organization*. 1994.

102. Rogers EM, Singhal A, Quinlan MM. Diffusion of innovations. InAn integrated approach to communication theory and research 2014 Apr 8 (pp. 432-448). Routledge.

103. Weiner BJ, Lewis CC, Stanick C, Powell BJ, Dorsey CN, Clary AS, Boynton MH, Halko H. Psychometric assessment of three newly developed implementation outcome measures. Implement Sci. 2017 Aug 29;12(1):108.

104. Wagner GJ, Wanyenze RK, Beyeza-Kashesya J, Gwokyalya V, Hurley E, Mindry D, Finocchario-Kessler S, Nanfuka M, Tebeka MG, Saya U, Booth M, Ghosh-Dastidar B, Linnemayr S, Staggs VS, Goggin K. "Our Choice" improves use of safer conception methods among HIV serodiscordant couples in Uganda: a cluster randomized controlled trial evaluating two implementation approaches. Implement Sci. 2021 Apr 15;16(1):41.

105. Barrington C, Rosenberg A, Kerrigan D, Blankenship KM. Probing the processes: longitudinal qualitative research on social determinants of HIV. AIDS and Behavior. 2021 Nov;25(2):203-13.

106. Palinkas LA, Aarons GA, Horwitz S, Chamberlain P, Hurlburt M, Landsverk J. Mixed method designs in implementation research. *Adm Policy Ment Health.* 2011;38(1):44-53.
